# Context Shapes (Proto)Conversations in the First Year of Life

**DOI:** 10.1111/desc.70018

**Published:** 2025-04-10

**Authors:** Zuzanna Laudańska, Karolina Babis, Agata Kozioł, Magdalena Szmytke, Peter B. Marschik, Dajie Zhang, Anna Malinowska‐Korczak, David López Pérez, Przemysław Tomalski

**Affiliations:** ^1^ Institute of Psychology, Polish Academy of Sciences Warsaw Poland; ^2^ Department of Child and Adolescent Psychiatry University Hospital Heidelberg Heidelberg University Heidelberg Germany; ^3^ Institute of Psychology Faculty of Philosophy and Social Sciences Nicolaus Copernicus University in Toruń Torun Poland; ^4^ iDN – Interdisciplinary Developmental Neuroscience, Division of Phoniatrics Medical University of Graz Graz Austria; ^5^ Center of Neurodevelopmental Disorders (KIND) Department of Women's and Children's Health Centre for Psychiatry Research, Karolinska Institutet & Region Stockholm Stockholm Sweden; ^6^ Child and Adolescent Psychiatry and Psychotherapy University Medical Center Göttingen, German Center for Child and Adolescent Health (DZKJ) and Leibniz Science Campus Primate Cognition Göttingen Germany

**Keywords:** infants, speech‐language development, turn‐taking, vocalizations

## Abstract

Speech development occurs in highly variable environments; however, little is known about the effect of situational context on emerging infant vocalizations. At 4 time points (4, 6, 9, and 12 months), we longitudinally measured vocalizations of 104 White infant‐caregiver dyads (41 girls) during three play contexts: book‐sharing, toy play, and rattle‐shaking. The frequency of infant vocalizations differed between contexts only at 12 months of age. Meanwhile, caregivers systematically spoke more frequently during book‐sharing than in other contexts from 4 months of age onwards. Book‐sharing elicited more conversational turns at the dyadic level than in other contexts from 9 months of age. Our results show emergence of vocal differentiation of play context by infants and the role of book‐sharing in facilitating early vocal turn‐taking.

## 1 Introduction

Language development largely happens in the context of social exchanges (e.g., Goldstein and Schwade 2008; Gros‐Louis et al. 2006, 2014) as infants actively participate in protoconversations from a very early age (e.g., Gratier et al. 2015; Harder et al. 2015; Hilbrink et al. 2015). More advanced speech‐like vocalizations (that contain syllables) happen more often during turn‐taking than when the infant is alone (Bloom et al. 1987; Long et al. 2022), highlighting the importance of social context for language learning. In the first 6 months of life, infants learn about the social efficacy of their vocalizations as they understand that the sounds they produce influence other people's actions (Goldstein et al. 2009; Elmlinger et al. 2023). Moreover, by vocalizing, infants elicit from their caregivers the production of shorter utterances and less lexically diverse contingent speech, thus they shape their own language environment (Elmlinger et al. 2019; Elmlinger et al. 2023; Elmlinger et al. 2025). On the other hand, caregivers’ temporally contingent feedback to infants’ babbling restructures its phonological patterns and elicits more complex vocal behavior (Goldstein et al. 2003; Goldstein and Schwade 2008). Altogether, vocalizations both influence and are influenced by interactions with social partners through the social feedback loop (e.g., Elmlinger et al. 2023; Goldstein and Schwade 2008; Warlaumont et al. 2014).

**Summary**

- Infants’ vocal production becomes context‐dependent only at 12 months of life when infants vocalize more during book‐sharing and rattle‐shaking than during playing with manipulative toys.
- The number of dyadic conversational turns differed between play contexts already from the age of 9 months, with the highest number during book‐sharing.
- Caregivers consistently speak more to their infants during book‐sharing than during rattle‐shaking and playing with manipulative toys between 4 and 12 months of infants’ age.

Conversations are not only situated socially but are also embedded in different settings and changing environments. However, when do infants begin to differentiate various situational contexts and types of activities? Very few studies investigated the role of situational factors in shaping infant vocal production across different ages (see Nguyen et al. 2022, for a discussion). Rome‐Flanders and Cronk (1995) showed that infants’ vocalizations were similar during peek‐a‐boo and play with a ball (measured between 6 and 24 months of age), whereas Sosa (2016) observed that infants aged 10–16 months vocalized more during play with books than with electronic or traditional toys. Similarly, Hsu et al. (2014) showed form–function decoupling between vocalization types across peek‐a‐boo and tickle games between 6 and 12 months of age (cross‐sectional study). These findings suggest that the second half of the first year of life might be the period of emerging task‐related differences with respect to vocal behaviors (Hsu et al. 2014; Sosa 2016). Altogether, there are inconsistent findings regarding the effects of varying situational contexts on infant vocal production and their approximate age of onset.

The second half of the first year of life is also the period of considerable gains in cognitive and motor skills that facilitate social interaction. Sustained attention (e.g., Amso and Scerif 2015), joint attention (e.g., Bakeman and Adamson 1984), as well as improvements in working memory (Reynolds and Romano 2016) all contribute to the advances in communication. Furthermore, the acquisition of independent sitting, standing, crawling, and walking are accompanied by increasing socio‐communicative skills (e.g., Adolph and Hoch 2019; Iverson 2010; Iverson 2021; Iverson 2022; Kretch et al. 2022; Luo and Franchak 2020; Schneider and Iverson 2022; Yamamoto et al. 2020; West and Iverson 2021). Different body postures allow the infant to free up their hands for more advanced types of object manipulation (e.g., Soska and Adolph 2014), which leads to a better understanding of the type of action that can be done with a particular set of objects and generates novel learning inputs. This could then drive the patterns of dyadic vocal communication and contribute to choosing the most appropriate actions in a given context.

Therefore, in this exploratory study, we longitudinally investigated the effect of varying situational play contexts on dyadic vocal interactions at four time points across the first year of life (at 4, 6, 9, and 12 months) to better understand when do infants and caregivers begin to differentiate various situational contexts. We investigated infant and caregiver vocal production and the number of conversational turns at the dyadic level in three types of infant‐parent play contexts: (a) book‐sharing, (b) playing with toys eliciting various manual actions (“manipulative toys”), and (c) rattle‐shaking. These three types of activities differ in task demands. Rattle‐shaking is considered the most constrained task, eliciting highly repetitive arm movements, which may be coupled with rhythmic vocalizations (Borjon et al. 2024). In contrast, playing with manipulative toys is considered the most free‐flowing interaction, focused on multimodal exploration of interesting toys, with variable arm and hand movements. Finally, book‐sharing elicits more visual and vocal than motor actions, as the benefits of book‐sharing for speech and language development were previously reported (e.g., Clemens and Kegel 2021; see review in Murray et al. 2022). The motor patterns during book‐sharing seem to be less clearly defined than in the two other tasks due to the more stationary character of this play type.

Previous research showed that patterns of infant limb movement become dependent on play context from 9 months of age (Laudańska et al. 2022). Based on this finding as well as overall developmental changes in the second half of the first year of life in motor, cognitive, and socio‐emotional skills, we predict task‐related differences in infants' vocalizations and dyadic vocal turn‐taking from around 9 months of age.

By contrast, based on previous literature on the benefits of book‐sharing for enriching caregivers’ child‐directed speech (Clemens and Kegel 2021; see also Murray et al. 2022), we predict that caregivers will consistently, across all measured time points, show a higher rate of vocal production during book‐sharing than during the two other tasks.

## 2 Methods

### 2.1 Participants

Participants included 104 infant‐parent dyads (41 girls) who were invited to the lab when infants were 4 (T1), 6 (T2), 9 (T3), and 12 (T4) months old (see Table 1). Eighty three dyads participated in a minimum of three visits, and out of them, 48 dyads contributed data at all 4 time points (missed visits are mostly due to COVID‐19‐related restrictions as data collection was conducted between 2020 and 2023). Participants were from predominantly middle‐class families (indicated by the socio‐economic data on income and education self‐disclosed by caregiver(s)) living in Warsaw, Poland (> 1.8 million inhabitants) and the surrounding metropolitan area (> 3 million inhabitants). The majority (90%) of the caregivers had completed higher education: 3 held a PhD degree, 81 held a master's degree, 10 held a bachelor's, and 4 completed high school (6 missing data). Overall, in 102 cases, the same caregiver interacted with the infant during all visits (101 mothers, 1 father). In two cases, because of the availability for scheduling an appointment, different parents interacted with the child during different time points. In some cases, two caregivers (both parents or a parent and a non‐parental caregiver) were present during the visit, but only one of them was in the testing room interacting with the child during recording. The other caregiver stayed in the adjacent room and interacted with the child only during breaks in the recording (e.g., during feeding).

**TABLE 1:** Sample characteristics.

Time point	Book‐sharing		Playing with manipulative toys		Rattle‐shaking	
	*N*	Mean age (SD); Range	*N*	Mean age (SD); Range	*N*	Mean age (SD); Range
T1	65	4.33 (0.26); 3.9–4.9	68	4.35 (0.26); 3.9–4.9	66	4.35 (0.26); 3.9–4.9
T2	76	6.63 (0.39); 6.0–7.8	75	6.62 (0.40); 6.0–7.8	77	6.62 (0.40); 6.0–7.8
T3	69	9.07 (0.38); 8.3–9.9	71	9.07 (0.38); 8.3–9.9	69	9.08 (0.38); 8.3–9.9
T4	68	12.14 (0.53); 11.5–13.5	72	12.12 (0.52); 11.5–13.5	71	12.13 (0.52); 11.5–13.5

Data from 4 participants were excluded due to preterm birth (*N* = 1), perinatal complications (*N* = 2), and family history of autism spectrum disorder (*N* = 1). In addition, data from 4 visits of another 4 participants were excluded due to failure to schedule visits within the planned age ranges (see Table 1 for a full overview of included participants at each time point and task). Thus, all included infants were reported by their caregivers to be typically‐developing. For their participation, infants received a diploma and a small gift. The study conformed to the Declaration of Helsinki and received clearance from the Research Ethics Committee at the Institute of Psychology of the Polish Academy of Sciences in Warsaw, Poland.

### 2.2 Procedure

Interactions were recorded in an infant‐friendly laboratory room on a carpeted play area. Upon the family's arrival, an experimenter explained the study protocol and obtained informed parental consent. Once the infant was familiarized with the laboratory, wearable motion trackers and head cameras were put on the infant and caregiver (data not reported here). Then, a set of parent‐child interaction tasks with different sets of age‐appropriate toys took place. The sets for infants aged 4 and 6 months were slightly different from those for infants aged 9 and 12 months to maintain their interest in a given task as well as to adjust the size and weight of objects to infants’ motor skills as well as advancements in overall infant development (see Lourenço et al. 2022, for a discussion on importance of adjusting the setup and materials to the infant's developmental level while studying vocal turn‐taking; see also Figure S1 for an illustration of the toys in each task and time point). There were 6–7 different tasks during each recording session, but here we report data comparing three of them: book‐sharing, playing with manipulative toys, and rattle‐shaking, each lasting around 5 min. For the present analyses, we have chosen three tasks that required qualitatively different actions—rhythmic body movements to produce the rattling sound, various reaching, holding, pushing, and pulling actions to explore manipulative objects, or more vocal actions during book‐sharing. The order of play activities was randomized between participants and testing sessions. There were no specific instructions to the caregivers, they were asked to play with their child as they usually would, using the provided objects.

All plays were recorded using three remote‐controlled HD CCTV cameras (Axis) and the sound recording synchronized with the video system was carried out with a high‐grade cardioid membrane condenser microphone (Sennheiser e914) placed underneath one of the cameras. Since some testing sessions were shortened due to the infant's fussiness (which resulted in omitting one or more of the interaction tasks) and the unavailability of audio data from several testing sessions due to technical problems with the microphone, Table 1 presents the number of dyads contributing data at every task and time point. In total, 76.16 h of infant‐parent interactions were annotated at two passes—separately for infant and parent vocal production. The total duration of each task at each time point is depicted in Figure S2. On average, the duration of playing with manipulative toys was slightly longer than book‐sharing and rattle‐shaking (Supporting Information: Analysis 2).

### 2.3 Book‐Sharing Task

In the book‐sharing task, the dyads were provided with several baby books. At T1 and T2, there were three small picture books: one with nursery rhymes, one with big pictures of animals and people, and one with pictures and onomatopoeic words. At T3 and T4, infants and parents were given one bigger book with pictures and onomatopoeic words and one smaller book with animal pictures, nursery rhymes about animals, and tactile elements. All books were provided at the same time and the dyads could choose freely which ones they used.

### 2.4 Manipulative Toys Task

In the manipulative toys task, infants and parents were given a set of toys that varied in tactile structure and provided multimodal feedback (sounds, movements). Two toys were the same at all time points: a sensory pop‐it toy and a gliding, rolling, and rattling sensory toy with tactile silicone elements. In addition, at T1 and T2, the set consisted of a wooden wiggly worm, a sensory toy with different tactile fabric and silicone elements, and a grasping ball with finger holes and rattling beads, whereas at T3 and T4: a spinning toy with small balls inside and a sensory‐exploration toy with elements with different textures that can be pushed, spun, or clicked and make different sounds. All toys were provided at the same time and the dyads could choose freely which ones they used.

### 2.5 Rattle‐Shaking Task

During a rattle‐shaking task, dyads received two maracas rattles and two rattles of different types: barbell rattles for younger infants and teddy bear rattles for older ones. All rattles were provided at the same time and the dyads could choose freely which ones they used.

### 2.6 Coding of Infant Vocalizations

For each interaction session, infants' vocalizations were coded off‐line using PRAAT software (Boersma and Weenink 2020). The coders (the first and second authors of this paper) marked the onsets and offsets of each vocalization at the utterance level. An utterance was defined as a vocalization occurring on one expiration cycle (Vihman et al. 1985; Nathani and Oller 2001). All prelinguistic speech‐like vocalizations were coded: protophones (squeals, vowel‐like sounds, growls, whispers, yells, grunts), syllables, and (proto‐)words (based on Buder et al. 2013 and Warlaumont et al. 2014; see Supporting Information for a full coding scheme). Reflexive sounds (laugh and cry) were not analyzed for the aims of the present study. Annotations were saved to TextGrid format files and descriptive data were extracted using in‐house MATLAB (2019b) script and mPraat toolbox (Bořil and Skarnitzl 2016). The descriptive data on the mean duration of vocalization are presented in Figure S3.

### 2.7 Coding of Parental Utterances

For each interaction session, parental speech was coded in a separate step (using the same software) by the same two coders as for infant vocalizations and 11 specifically trained students. In coding the utterances produced by the caregiver, four coding categories were formed: laughter, speech, vocalization, and singing. If the silent pause following the vocal sound was greater than 200 ms, two successive sounds were coded (Harder et al. 2015). Laughing was excluded from the analyses, whereas speaking, vocalizing, and singing were considered as “caregiver's vocal production” in further analyses. Descriptive data were extracted analogously to the infant vocalizations. The descriptive data on the mean duration of utterance are presented in Figure S4.

### 2.8 Inter‐Rater Agreement

The coders were primarily assigned to individual infants and caregivers on a mostly random basis. Efforts were made to avoid systematic assignment of the same coder to the same caregiver or infant across multiple visits. However, in some cases, the same coder may have been assigned to code the same type of task across different age points for different infants. This approach aimed to reduce potential coder variability by distributing coding responsibilities evenly and avoiding consistent coder‐infant/caregiver pairing over time.

To calculate inter‐rater agreement, ∼10% (100 files for parental utterances, 86 for infant vocalizations) of recordings in each task and time point were double‐coded (randomized across coders for parental utterances). Cohen's kappa was 0.82 for parental utterances and 0.85 for infant vocalizations.

### 2.9 Turn‐Taking Analysis

**FIGURE 1:**
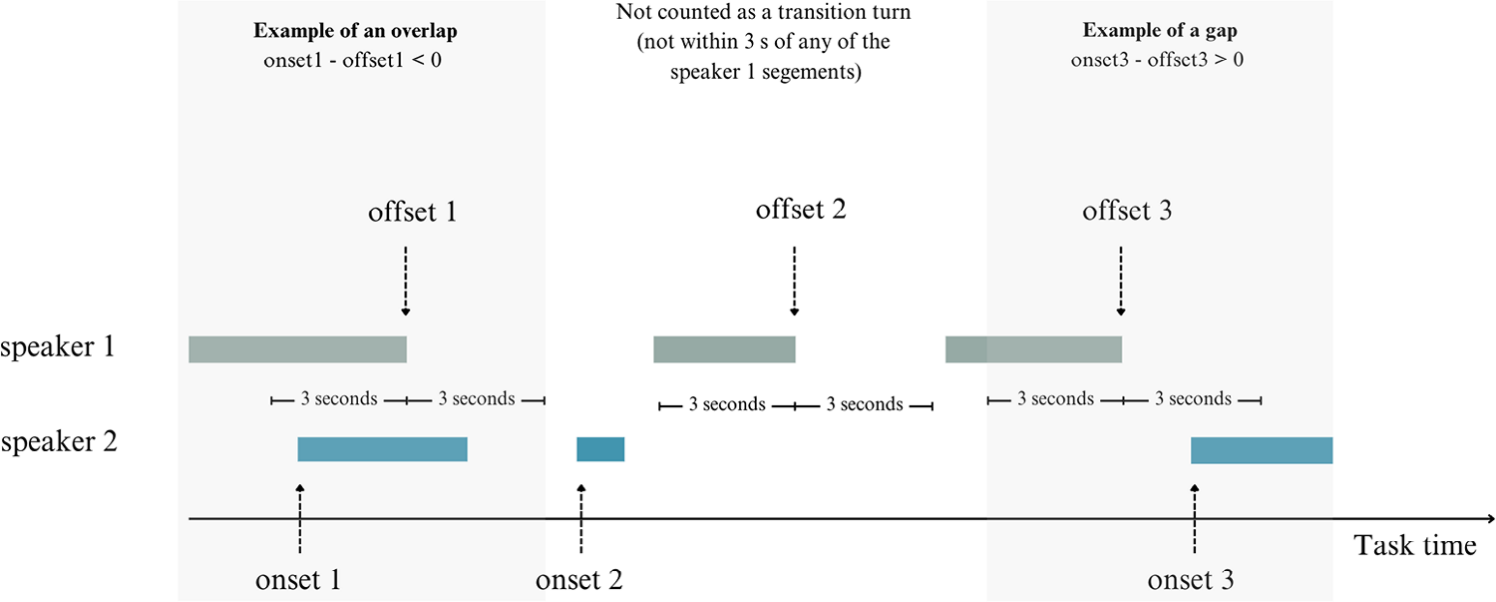
Calculation of dyadic vocal coordination (conversational turns).

To calculate the number of conversational turns, we adapted a Python script from Trujillo and Pouw (2021) and Trujillo et al. (2021), using *parselmouth* (Jadoul et al. 2018), *praatio, pympi*, and *tabulate* libraries. Based on the annotations, we calculated the number of conversational turns from the perspective of an infant by taking the offset of each vocalization of an infant and finding thereafter the onset of the nearest caregiver's vocalization within a window of 3 s (as in Gratier et al. 2015 Harder et al. 2015; Nguyen et al. 2022). Turn transition time was first calculated as the time difference between the caregiver's onset and the infant's offset (see Figure 1). If the caregiver began speaking before the infant finished vocalizing, the turn transition time was negative, indicating an overlap. In contrast, positive values indicated gaps between speakers. In the second step, we ran the reversed analysis—the time difference between the infant's onset and the caregiver's offset.

### 2.10 Statistical Analyses

To assess the repeated‐measures effects of time point (4) and task (3), we ran the General Estimating Equations (GEEs) with a Bonferroni correction for pairwise comparisons for each outcome variable separately. GEE is a robust modeling technique particularly suited to longitudinal data as it acknowledges that measurements from the same infants are not independent across different time points. It is a non‐parametric method, requiring neither independence nor normality assumptions, and it follows a conservative approach to handle missing data, which helps ensure the robustness of the findings (for previous application of this approach in investigating early infant vocalizations see Long et al. 2022, Long et al. 2024; Oller et al. 2024). Furthermore, as a comparison, we also present the results of the analysis of the Maximum Likelihood (ML) linear mixed‐effect models in the Supporting Information.

### 2.11 Transparency and Openness

The data that support the findings will be available upon request from the corresponding authors following an embargo period from the date of publication to allow for the finalization of the ongoing longitudinal project. The analytic code is publicly accessible at the following URL: https://osf.io/m6xkw/?view_only=2fb2e78c38aa4b3a8e22cf8e92d04cdf. The statistical analysis was run in R (R Core Team 2023) version 4.3.1 (2023‐06‐16) and RStudio (2023.06.0+421) using *tidyverse, geepack* (Halekoh et al. 2006), *lme4* (Bates et al. 2015), and *emmeans* (Lenth 2025) libraries and visualized using *ggplot2* (Wickham 2016). The analyses were not pre‐registered.

## 3 Results

### 3.1 Rate Per Minute of Infant Vocalizations

**FIGURE 2:**
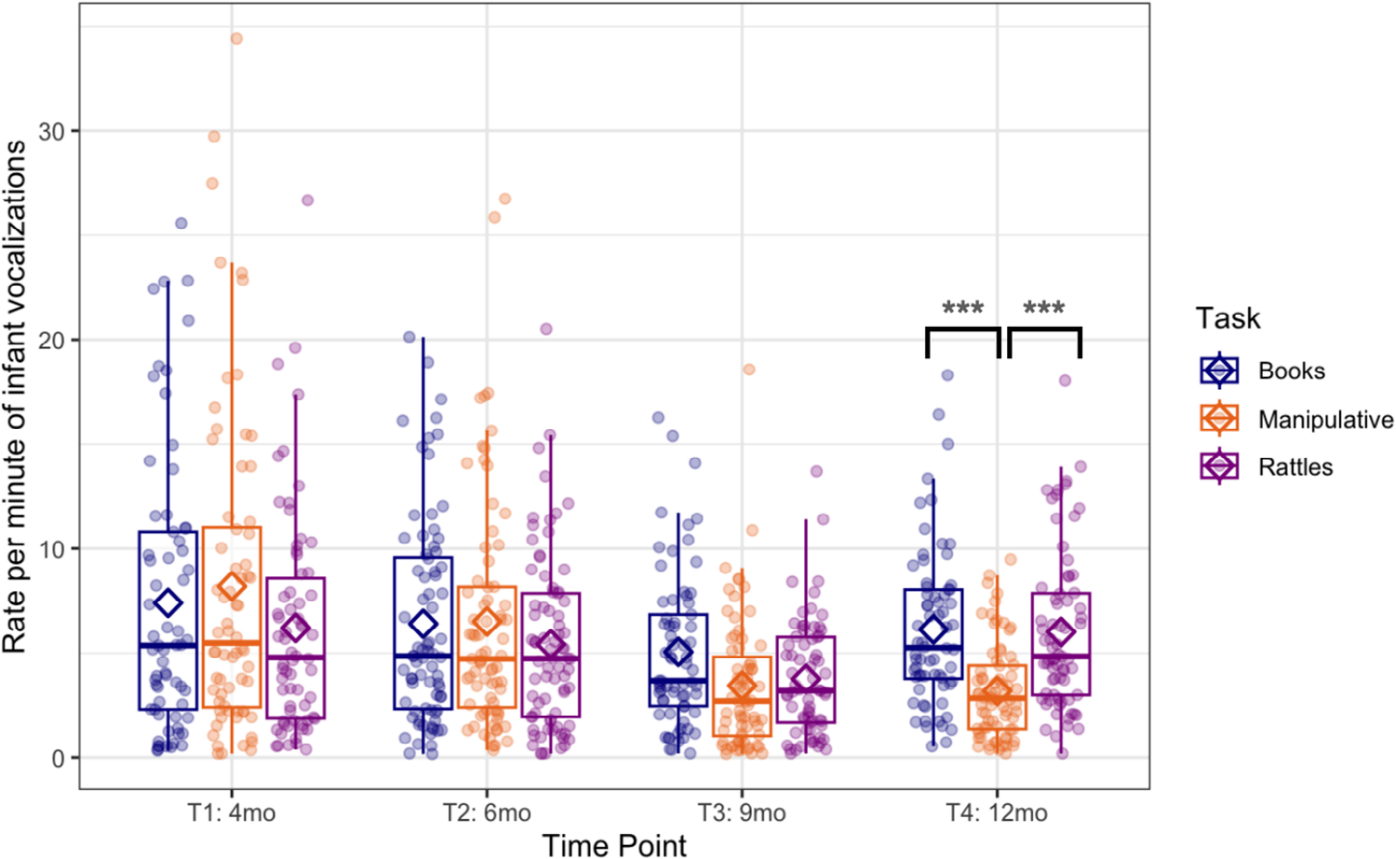
Boxplots showing frequency (rate per minute) of infant vocalizations at each time point during book‐sharing (blue), playing with manipulative toys (orange), and rattle‐shaking (purple). Horizontal lines represent the median value, boxes are drawn from the first quartile to the third quartile, and whiskers indicate min and max values. Diamonds represent mean scores. Significant differences indicated by asterisks: *** *p* < 0.001.

The GEE with time point (4) and task (3) as within‐subjects factors showed a significant difference in rate per minute of infants’ vocalizations between tasks (Wald *χ* ^2^(2) = 6.7, *p* = 0.034; see Figure 2) and time point (Wald *χ* ^2^(3) = 51.3, *p* < 0.001), as well as the interaction effect (Wald *χ* ^2^(6) = 30.2, *p* < 0.001). Post hoc pairwise comparisons revealed that there were no task‐related differences at T1, T2, and T3. At T4, infants vocalized more during book‐sharing than during play with manipulative toys (*p* < 0.001) and more during rattle‐shaking than play with manipulative toys (*p* < 0.001). The difference between book‐sharing and rattle‐shaking was not significant.

### 3.2 Rate Per Minute of Caregiver Vocal Production

**FIGURE 3:**
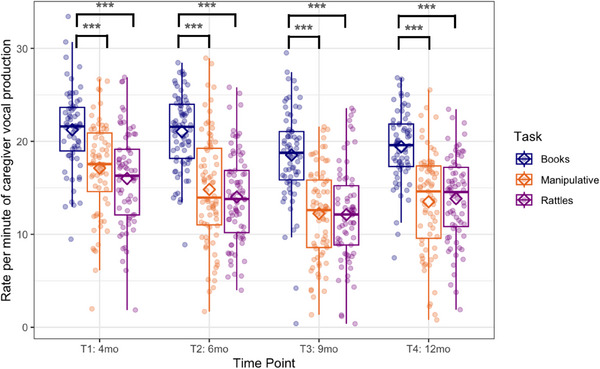
Boxplots showing frequency (rate per minute) of caregiver vocal production at each time point during book‐sharing (blue), playing with manipulative toys (orange), and rattle‐shaking (purple). Horizontal lines represent the median value, boxes are drawn from the first quartile to the third quartile, and whiskers indicate min and max values. Diamonds represent mean scores. Significant differences indicated by asterisks: *** *p* < 0.001.

The GEE with time point (4) and task (3) as within‐subjects factors showed the main effects of time point (Wald *χ* ^2^(3) = 54.1, *p* < 0.001; see Figure 3) and task (Wald *χ* ^2^(2) = 308.6, *p* < 0.001) in the rate per minute of the caregiver's speaking. Caregivers were speaking more frequently during the book‐sharing task than during the other two tasks (both *ps* < 0.001). The interaction effect was not significant (Wald *χ* ^2^(6) = 7.1, *p* = 0.31). The difference between rattle‐shaking and playing with manipulative toys was not significant.

## 4 Dyadic Vocal Coordination

### 4.1 Rate Per Minute of Conversational Turns

**FIGURE 4:**
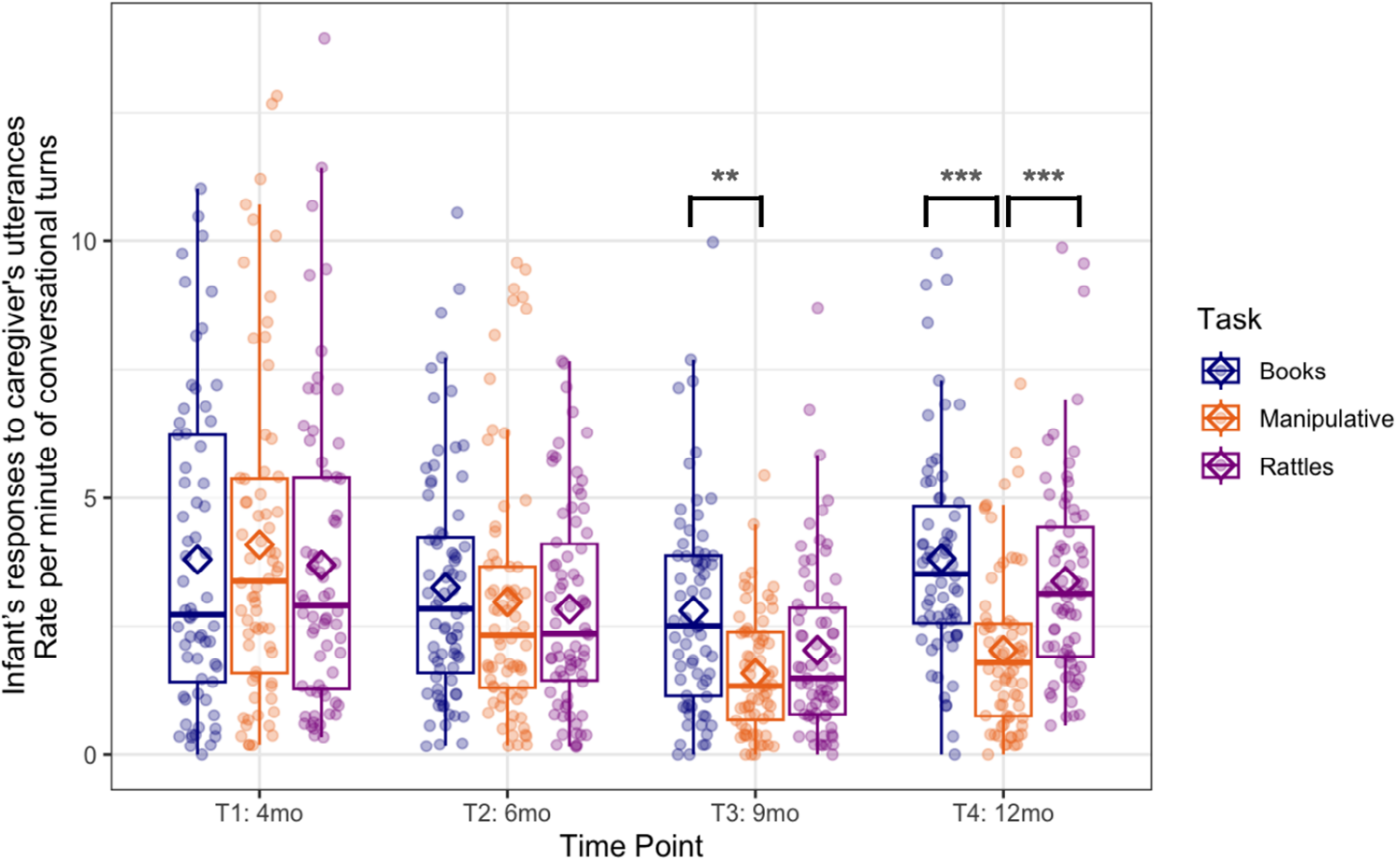
Boxplots showing the infant's responses to the caregiver's utterances calculated as a rate per minute of conversational turns at each time point during book‐sharing (blue), playing with manipulative toys (orange), and rattle‐shaking (purple). Horizontal lines represent the median value, boxes are drawn from the first quartile to the third quartile, and whiskers indicate min and max values. Diamonds represent mean scores. Significant differences indicated by asterisks: *** *p <* 0.001; ** *p <* 0.01; * *p <* 0.05.

When infant's responses to caregiver's utterances were analyzed (caregiver as Speaker 1 in Figure 1; infant as a responding person), the GEE with time point (4) and task (3) as within‐subject factors showed the main effects of time point (Wald *χ* ^2^(3) = 60.3, *p* < 0.001) and task (Wald *χ* ^2^(2) = 14.5, *p* < 0.001) as well as a significant interaction effect (Wald *χ* ^2^(6) = 21.3, *p* < 0.001) in the rate per minute of conversational turns (see Figure 4). At T3 there were more conversational turns during book‐sharing than play with manipulative toys (*p* = 0.001). The difference between rattle‐shaking and book‐sharing or playing with manipulative toys was not significant. At T4, there were fewer conversational turns during play with manipulative toys than during book‐sharing (*p* < 0.001) and rattle‐shaking (*p* < 0.001). There were no significant differences between book‐sharing and rattle‐shaking at T4. Importantly, there were no significant task differences at the first two time points, which shows the progressive emergence of specialization to task demands across the first year of life.

**FIGURE 5:**
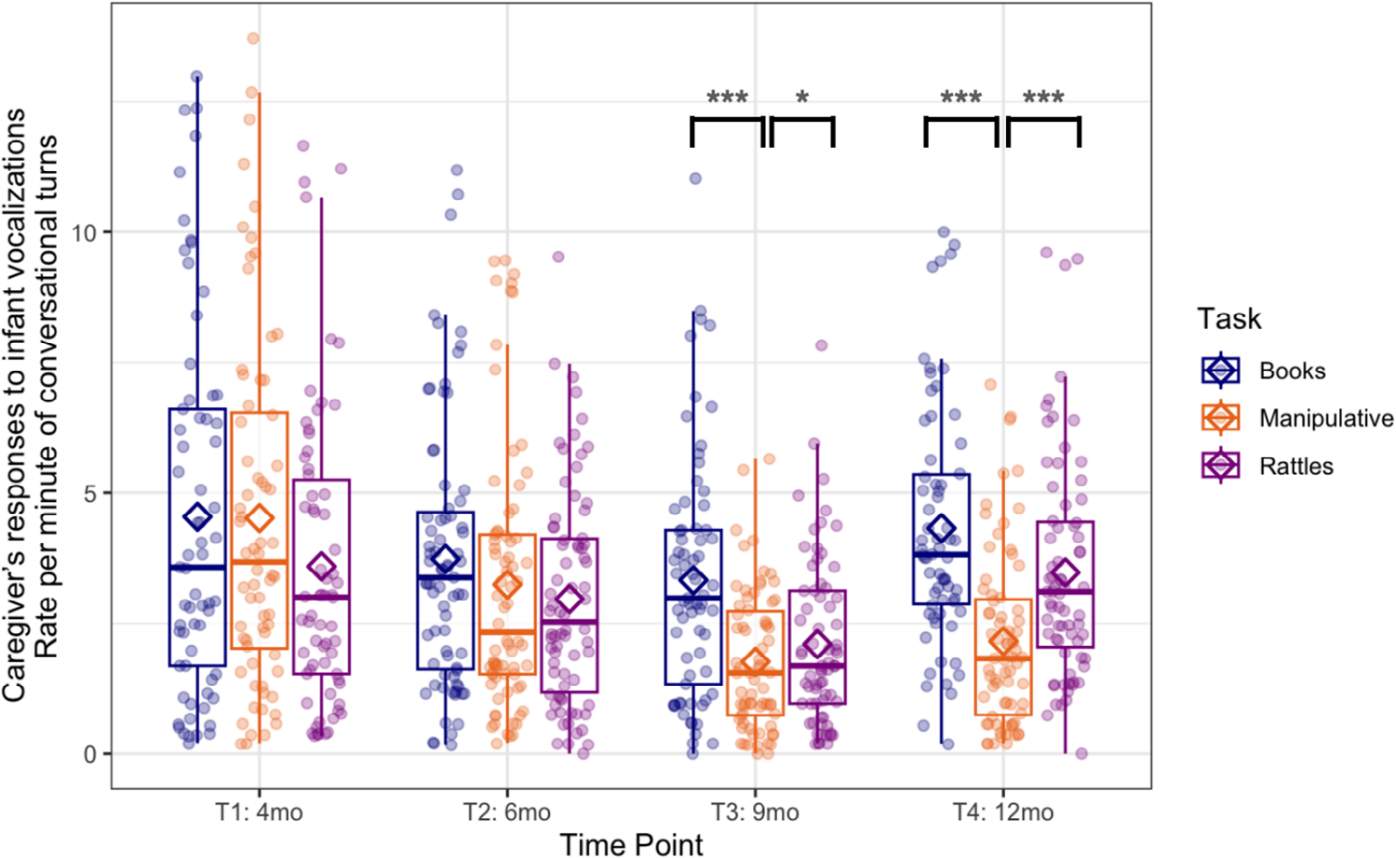
Boxplots showing caregiver's responses to infant's utterances calculated as a rate per minute of conversational turns at each time point during book‐sharing (blue), playing with manipulative toys (orange), and rattle‐shaking (purple). Horizontal lines represent the median value, boxes are drawn from the first quartile to the third quartile, and whiskers indicate min and max values. Diamonds represent mean scores. Significant differences indicated by asterisks: *** *p <* 0.001; * *p* < 0.05.

When the caregiver's responses to infant vocalizations were analyzed (infant as Speaker 1 in Figure 1; caregiver as a responding person), the GEE showed the main effects of time point (Wald *χ* ^2^(3) = 55.1, *p* < 0.001; see Figure 5), task (Wald *χ* ^2^(2) = 27.6, *p* < 0.001), as well as a significant interaction effect (Wald *χ* ^2^(6) = 23.3, *p* < 0.001). At T3, there were fewer conversational turns during play with manipulative toys than during book‐sharing (*p* < 0.001) and rattle‐shaking (*p* = 0.017). At T4, there were fewer conversational turns during play with manipulative toys than during book‐sharing (*p* < 0.001) and rattle‐shaking (*p* < 0.001). There were no significant differences between book‐sharing and rattle‐shaking at T4.

### 4.2 Mean Turn Transition Time (Gaps and Overlaps)

**FIGURE 6:**
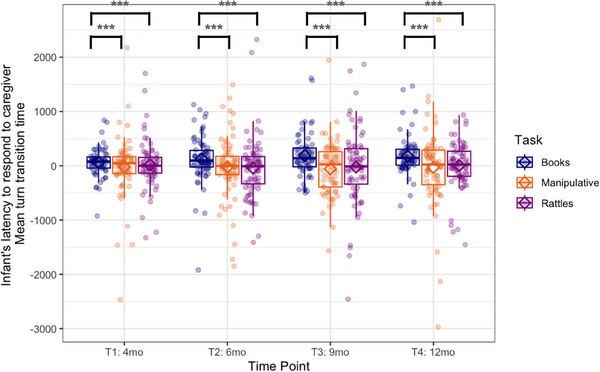
Boxplots showing the infant's latency to caregiver's utterances calculated as mean turn transition time at each time point during book‐sharing (blue), playing with manipulative toys (orange), and rattle‐shaking (purple). Positive values indicate gaps and negative overlaps. Horizontal lines represent the median value, boxes are drawn from the first quartile to the third quartile, and whiskers indicate min and max values. Diamonds represent mean scores. Significant differences indicated by asterisks: *** *p* < 0.001.

The GEE showed that, when the infant's responses to the caregiver's utterances were analyzed (caregiver as Speaker 1 in Figure 1), the mean turn transition time was task‐dependent (the latency of the infant's responses to the caregiver's utterances). The GEE showed a main effect of task (Wald *χ* ^2^(2) = 23.76, *p* < 0.001, see Figure 6), with higher values during book‐sharing than play with manipulative toys (*p* < 0.001) and rattle‐shaking (*p* < 0.001), and the mean value was above zero, indicating more gaps than overlaps. There was no significant difference between playing with manipulative toys and rattle‐shaking. Neither the effect of time point (Wald *χ* ^2^(3) = 0.44, *p* = 0.93) nor the interaction effect (Wald *χ* ^2^(6) = 4.30, *p* = 0.64) was significant.

**FIGURE 7:**
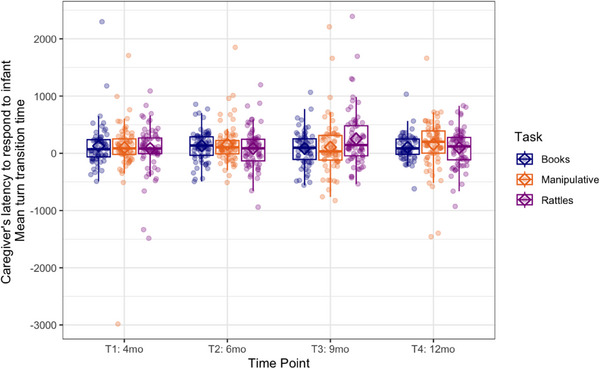
Boxplots showing caregiver's latency to infant's vocalizations calculated as mean turn transition time at each time point during book‐sharing (blue), playing with manipulative toys (orange), and rattle‐shaking (purple). Positive values indicate gaps and negative overlaps. Horizontal lines represent the median value, boxes are drawn from the first quartile to the third quartile, and whiskers indicate min and max values.

For the caregiver's responses to infant vocalizations, the mean turn transition time was not task‐ or age‐dependent. The latency of the caregiver's responses to the infant's vocalizations did not depend on the type of activity or the infant's age (see Figure 7). The GEE showed no significant effects of task (Wald *χ* ^2^(2) = 0.49, *p* = 0.78), time point (Wald *χ* ^2^(3) = 1.20, *p* = 0.78), or the interaction (Wald *χ* ^2^(6) = 8.32, *p* = 0.22), which indicates a constant level of response latency on the caregiver's side.

## 5 Discussion

In this study, we investigated the effect of age and situational context on the vocal production of both infants and caregivers across the first year of an infant's life. In particular, we studied at what age infants' and caregivers' vocal production and turn‐taking exchanges begin to differ depending on task demands (book‐sharing, playing with manipulative toys, rattle‐shaking). Overall, we observed that task‐related differences in infant vocalizations emerge around 12 months of age. Infants vocalized more during book‐sharing and rattle‐shaking than during playing with manipulative toys. In contrast, parents' verbal input was characterized by consistent task‐related differences from the first measured time point (at 4 months of an infant's age onwards). Not surprisingly, during all visits, parents engaged in more frequent vocal production during book‐sharing than during the two other tasks.

Finally, we investigated how the situational context affects dyadic verbal interactions. The number of conversational turns became task‐dependent already at T3. There were more dyadic exchanges during book‐sharing than playing with manipulative toys at 9 months and fewer dyadic exchanges during play with manipulative toys than book‐sharing and rattle‐shaking at 12 months. The fact that we observed differences related to situational play context in dyadic turn‐taking earlier in development (already at 9 months) than in individual measures of infant vocal production (observed only at 12 months) is particularly interesting. One possible explanation could be that dyadic interactions drive communicative development (e.g., Donnelly and Kidd 2021; Huber et al. 2023)—thus, the differences related to play activities are observed earlier in social reciprocity than in an individual‐focused analysis. We have also observed that the infant's mean turn transition time had higher positive values, indicating more gaps than overlaps in dyadic turn‐taking during book‐sharing than in two other activities. This effect was not dependent on the time point, which may suggest that book‐sharing supports an infant's timing of conversational turns across infancy. We have not found a similar pattern in caregiver's mean turn transition time, suggesting that their reaction time of responding vocally to their infant's vocalizations was constant, and not dependent on task or time point.

Lourenço et al. (2022) also showed differences in vocal turn‐taking between contexts. They observed that the frequency of conversational turns was higher in free play without toys than in dyadic play with toys at 7 and 12 months. Furthermore, they showed different developmental trajectories in relation to the timing of vocal turn‐taking across these two play contexts. During play with toys, there were no age‐related differences in turn transition time between 7 and 12 months. In contrast, during free play, there was a developmental decrease in infant gap duration. The authors’ conclusion that interactions without objects elicit shorter gap durations seems consistent with our analysis. Indeed, one of the benefits of book‐sharing activity may be related to eliciting longer gaps (fewer overlaps), which promotes the organization of early conversations when one person listens while the other person speaks. Further comparisons can be drawn with studies such as Hilbrink et al. (2015), which examined temporal coordination in infant‐caregiver vocal interactions. Their findings highlighted that infants' gap duration increased between 5 and 9 months and then slowly decreased between 12 and 18 months. Our results show that the type of situational context during which the protoconversation is happening may also have some effect on the timing of infant responses.

Our results support the multimodal approach to studying speech‐language development (Schroer et al. 2023), acknowledging the intertwinement of developmental domains. Verbal functioning (e.g., Oller 2010), gross motor development (e.g., Iverson 2010), and socio‐cognitive changes, as well as parental verbal input and responsiveness, jointly shape advances in communicative development. Interestingly, a similar developmental pattern of specialization to different play contexts was also observed in the motor domain. Infants’ limb movements were recorded during parent‐child interactions with wearable motion trackers and analyzed using multidimensional recurrence quantification analysis. The complexity (entropy) and dynamic stability (mean line of the recurrence plot) of limb movements became task‐specific at 9 months of age (Laudańska et al. 2022; Arellano‐Véliz et al. 2025). This suggests that the period of late infancy may be a window of intense and complex reorganization of actions across modalities. Emerging gross and fine motor skills that change the affordances for interactions with objects and social partners enable an increase in specialization to task demands. Such a reorganization at the level of an individual (within‐infant coordination) could also affect the between‐person coordination—for example, Suttora and Salerni (2011) found in preterm infants that their motor skill maturation works as a signal for mothers to modify and adjust their linguistic interactive style to the increased skills. Furthermore, in another modality—maternal touch—Serra et al. (2025) observed that maternal touch behaviors varied with the infant's age and the complexity of the interaction task, showing another way in which mothers modulate their actions to the infant's evolving needs.

Speech production is a highly complex motor action, requiring the coordination of multiple articulators. It is also accompanied by arm movements that, in adults, form a speech‐gesture system (e.g., Pouw and Fuchs 2022). According to Iverson and Thelen (1999), infants' coupling between gestures and speech is rooted in the oscillations between orofacial and arm movements. They proposed that the production of repetitive, rhythmically organized movements entrains vocal activity. Pouw and Fuchs (2022) proposed a revision of this idea, arguing that the exploratory limb movements that co‐occur with vocalizations affect these vocal productions through respiration. Our results that show a high rate per minute of infant vocalizations during rattle‐shaking at 12 months of age are in line with this theoretical proposal. Similarly, Borjon et al. (2024) showed evidence that hand and head movements co‐activate with spontaneous vocalizations during a tabletop dyadic play, further supporting the idea of motor‐vocal coupling. Overall, it seems that vocal‐motor babbling can be rooted in the biomechanical links between upper limb movements, postural muscles, and the respiratory system, but future research should investigate it further.

Furthermore, the results presented here provide additional support to the claim that dyadic book‐sharing activities are beneficial for communicative development already in infancy. We show that vocal turn‐taking happens more often during book‐sharing than during the two other types of infant‐parent play already from 9 months of age—so from the time when infants become able to pay attention to common referents (Trevarthen and Hubley 1978; Tronick et al. 1979; Abney et al. 2020). During book‐sharing, parents talked significantly more to their infants at all time points, already from the age of 4 months onwards. This remarkable consistency is in line with previous results by Rossmanith et al. (2014), who showed that book‐sharing interactions occurred from as early as 3 months, but their quality and dynamics changed together with the development of infants' motor skills and attention. Similarly, Sosa (2016) found that the frequency of infant (aged 10–16 months) vocalizations, adult words, and conversational turns was higher during book‐sharing than playing with traditional or electronic toys. Thus, our results suggest that already from the age of 4 months, infants may benefit from increased parental vocal input during book‐sharing activities.

The development of early vocalizations and turn‐taking exchanges with respect to the choice of stimuli and situational contexts may be important for understanding and interpreting developmental trajectories of pre‐linguistic development and intervention planning. More frequent turn‐taking face‐to‐face during infant‐parent interactions at 4–6 months of age was shown to be related to interpersonal neural synchrony, highlighting the importance of emerging turn‐taking for a child's brain and language development (e.g., Huber et al. 2023; Nguyen and Zimmer et al. 2023). The number of infants’ conversational turns has also been shown to predict language outcomes (e.g., Gilkerson et al. 2017; Romeo et al. 2018; Donnelly and Kidd 2021). Throughout infancy, structured interactions support the development of brain systems and pave the way for becoming a competent language user (e.g., Huber et al. 2023).

Findings from this study map onto interventional approaches focusing on parent‐child turn‐taking to build language skills at different linguistic levels (Ferjan Ramírez et al. 2020; McGowan et al. 2024). It adds to understanding the optimal timing of task‐specific interventions as well as age‐specific phenomena in the light of environmental changes (i.e., types of stimuli to engage in proto‐conversation). Learning regularities within parent‐child interaction loops and how infants discriminate and learn from social signals have to be seen in the light of perceptual integration (Mason et al. 2019) and vocal‐motor coordination. Atypical vocal production and motor‐vocal coupling in infancy may be a promising marker for the detection of infants at risk of developmental disorders such as autism spectrum disorder, Rett syndrome, and fragile X syndrome (e.g., Roche et al. 2018; Lang et al. 2019; Iverson and Wozniak 2007; Tenenbaum et al. 2020). Our results suggest that the situational play context should be taken into account when planning early interventions focused on vocal development.

Our longitudinal design, which consisted of short infant‐parent interactions with controlled sets of toys, allowed us to manually annotate every vocalization of both infants and caregivers and observe the emergence of task‐related differences in the first year of life. However, future studies should investigate whether a similar pattern of contextually situated proto‐conversations could also be observed in naturalistic daylong conversations with the annotated situational context of those exchanges. Future research should also investigate the potential cascading effects of motor development on vocal communication more directly by exploring the role of gross and fine motor development, postural stability, motor control, and the speech‐gesture system in more detail.

## 6 Limitations

A key limitation of this study lies in the semi‐structured nature of the tasks and the short duration of observations, which may not fully capture the dynamic and complex nature of infant‐caregiver turn‐taking in more naturalistic settings. The controlled environment, while valuable for standardization, may limit the generalizability of our findings to everyday interactions. Additionally, variability in caregiver behavior—potentially influenced by factors such as individual interaction styles and personality—may have introduced confounds, complicating the interpretation of results. This issue is particularly salient given that almost all caregivers in the study were mothers, leaving the pattern of turn‐taking with fathers unclear. Finally, the relatively small sample size presents a potential power issue, emphasizing the need for future research with larger, more diverse cohorts to draw more definitive conclusions about infant‐caregiver vocal interactions across the first year of life.

## 7 Conclusion

Infants’ vocal production becomes dependent on situational play context towards the end of the first year of life. Infants spent more time vocalizing during the book‐sharing and rattle‐shaking than playing with toys that elicit different manual actions at 12 months of age but not earlier. By contrast, caregivers consistently spoke more frequently to infants during book‐sharing than during the two other tasks at all measured time points. Importantly, the number of dyadic conversational turns differed between those play contexts already from the age of 9 months, with the highest number of turns during book‐sharing. Our results highlight differential patterns of vocal production across situational play contexts for both infants and caregivers. This adds to the understanding of the complexity of the speech‐language acquisition process, showing the importance of an age‐dependent role of context.

## Author Contributions

**Zuzanna Laudańska**: conceptualization, data curation, formal analysis, investigation, project administration, software, visualization, writing – original draft, writing – review and editing. **Karolina Babis**: data curation, investigation, project administration, writing – review and editing. **Agata Kozioł**: software, writing – review and editing. **Magdalena Szmytke**: writing – review and editing. **Peter B. Marschik**: writing – review and editing. **Dajie Zhang**: writing – review and editing. **Anna Malinowska‐Korczak**: Investigation, project administration, writing – review and editing. **David López Pérez**: conceptualization, software, writing – review and editing. **Przemysław Tomalski**: conceptualization, funding acquisition, project administration, supervision, writing – original draft, writing – review and editing.

## Conflicts of Interest

The authors declare no conflicts of interest.

## Supporting information



Supporting Information

## Data Availability

The data that support the findings will be available upon request from the corresponding authors following an embargo period from the date of publication to allow for the finalization of the ongoing longitudinal project. The analytic code is publicly accessible at the following URL: https://osf.io/m6xkw/?view_only=8e0d8838b85c44839b4e56a3d94f164e.
