# Supplementary material for: Context Shapes (Proto)Conversations in the First Year of Life
Source: Dev Sci. 2025 Apr 10;28(3):e70018. doi: 10.1111/desc.70018 (PMC11984065; doi:10.1111/desc.70018)
Supplement: Supplementary file 1 — Supporting Information [file DESC-28-e70018-s001.docx]

**Supplementary Information**

**Context shapes (proto)conversations in the first year of life**

Zuzanna Laudańska^1,3^, Karolina Babis^1^, Agata Kozioł^1^, Magdalena Szmytke^2^, Peter B. Marschik^3,4,5,6^, Dajie Zhang^3,4^, Anna Malinowska-Korczak^1^, David López Pérez^1^, Przemysław Tomalski^1^

^1^Institute of Psychology, Polish Academy of Sciences

^2^Institute of Psychology, Faculty of Philosophy and Social Sciences, Nicolaus Copernicus University in Toruń, Poland

^3^Department of Child and Adolescent Psychiatry, University Hospital Heidelberg, Heidelberg University, Heidelberg, Germany

^4^iDN – interdisciplinary Developmental Neuroscience, Division of Phoniatrics, Medical University of Graz, Austria

^5^Center of Neurodevelopmental Disorders (KIND), Department of Women’s and Children’s Health, Centre for Psychiatry Research, Karolinska Institutet & Region Stockholm, Stockholm, Sweden

^6^Child and Adolescent Psychiatry and Psychotherapy, University Medical Center Göttingen, German Center for Child and Adolescent Health (DZKJ) and Leibniz ScienceCampus Primate Cognition, Göttingen, Germany

1. **Pictures of the set of toys used on each task**


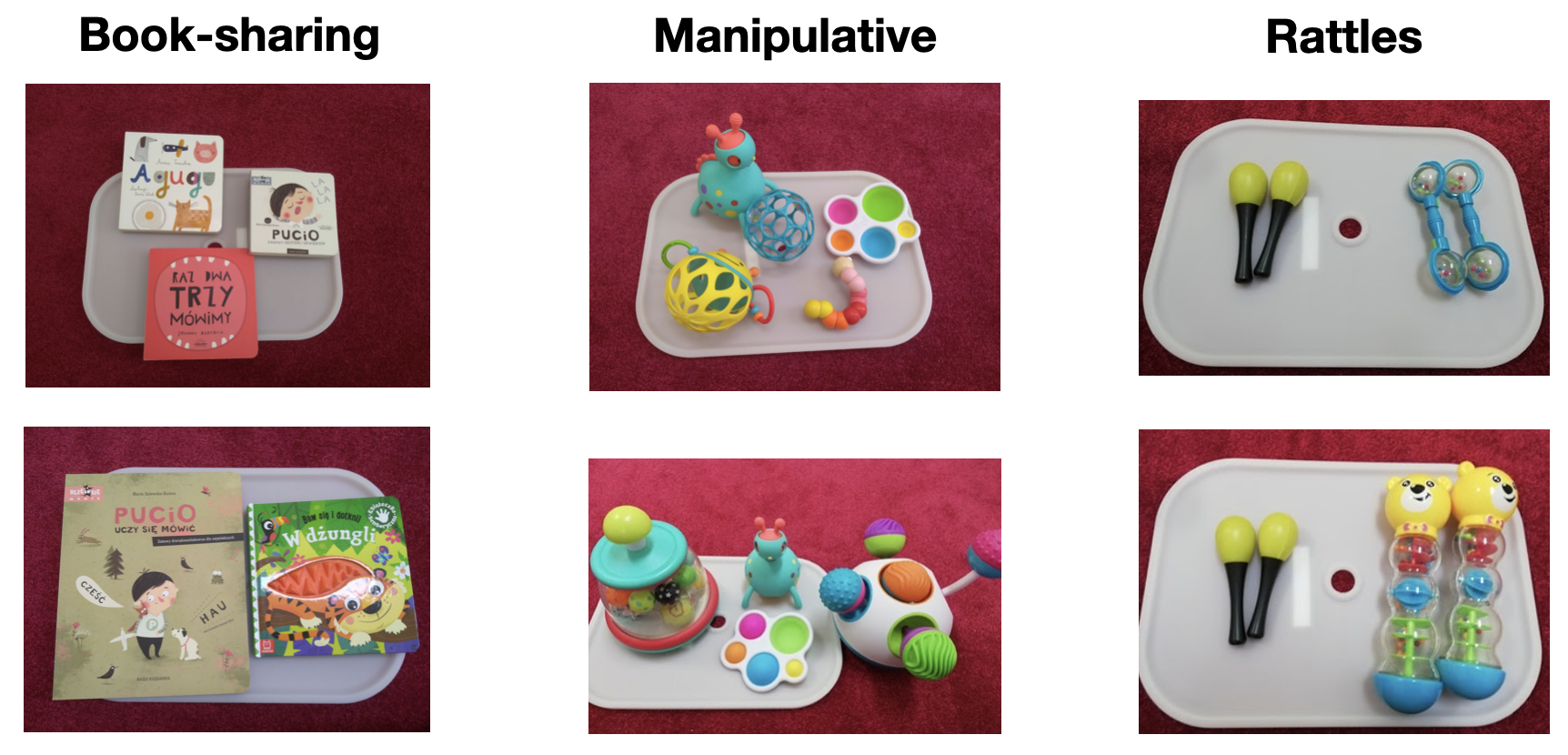


Fig. S1 The sets of toys used for each play. The top row indicates toys used during the visits at 4-6 months, and the bottom row indicates toys used at 9-12 months.

1. **Task Duration**

The GEE with time point (4) and task (3) as within-subjects factors showed the main effects of task (Wald χ2 (2) = 14.08, p < 0.001 in the duration of the task. The duration of playing with manipulative toys was longer than the duration of book-sharing (p < 0.001) and rattle-shaking (p < 0.001). Neither the effect of time point (Wald χ2(3) = 4.41, p = 0.220) nor the interaction effect (Wald χ2(6) = 3.63, p = 0.727) were significant.


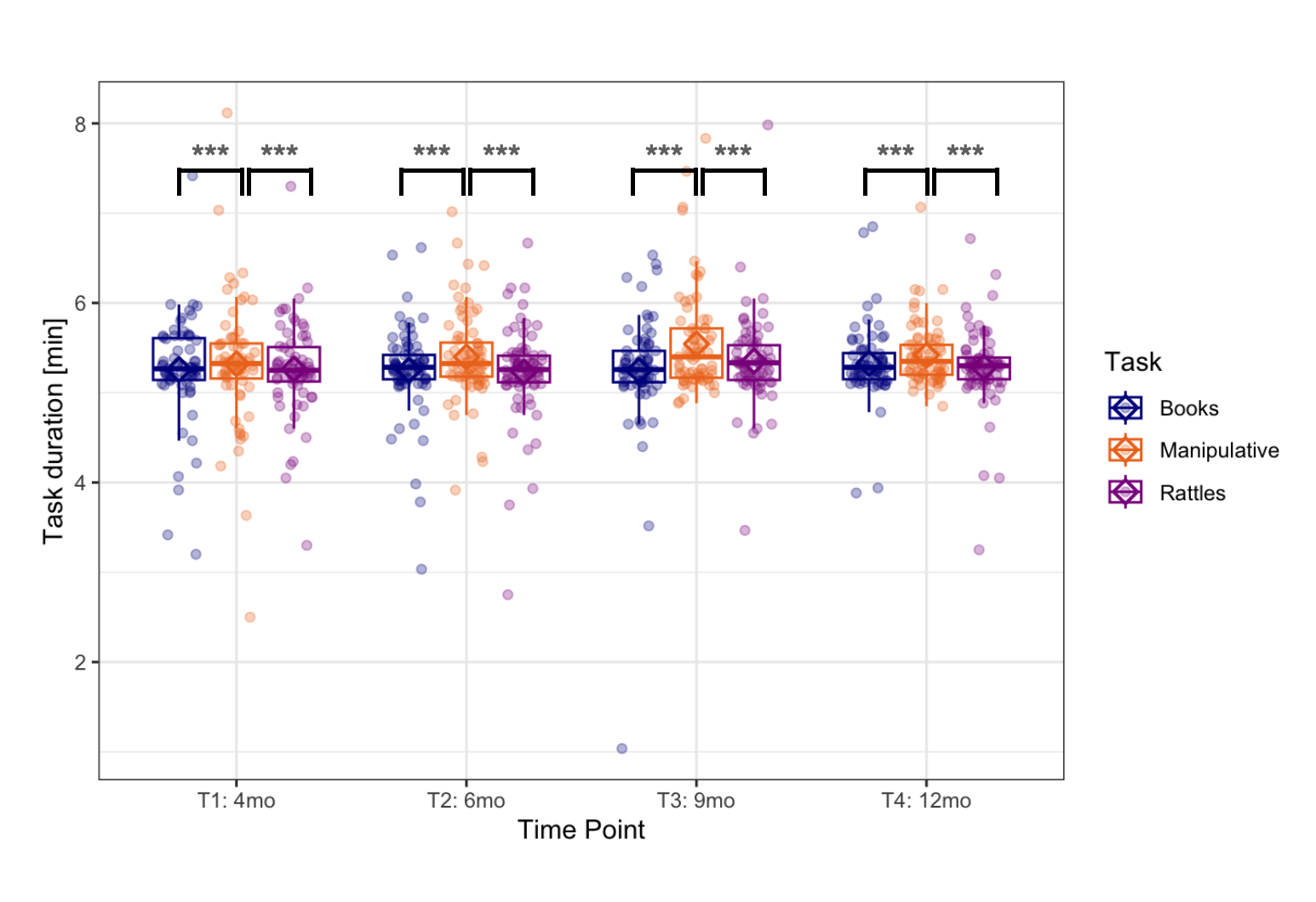


Fig. S2 Boxplots showing the duration of a task at each time point during book-sharing (blue), playing with manipulative toys (orange), and rattle-shaking (purple). Horizontal lines represent the median value, boxes are drawn from the first quartile to the third quartile, and whiskers indicate min and max values. Diamonds represent mean scores. Significant differences indicated by asterisks: ***, *p < .001.*

1. **Coding scheme**

**3.1. Coding of infant vocalizations**

All prelinguistic speech-like vocalizations were classified into four distinct, non-overlapping categories (based on Buder et al., 2013): a) reflexive sounds (laugh and cry), b) protophones such as squeals, vowel-like sounds, growls, whispers, yells, grunts; c) syllables, and d) words. Reflexive sounds (laugh and cry) were excluded from further analyses, whereas protophones, syllables, and words were considered „speech-related vocalizations” (Warlaumont et al., 2014) in further analyses.

**3.2. Coding of parental utterances**

Speech was defined as the production of words (containing at least one syllable) and phrases. Vocalization was defined as the production of vocal sounds that did not contain syllables. Singing was coded if parents produced musical tones (both singing and humming). These three categories were mutually exclusive (e.g., singing a song containing words would be coded as singing).

1. **Mean duration of vocalization or utterance**


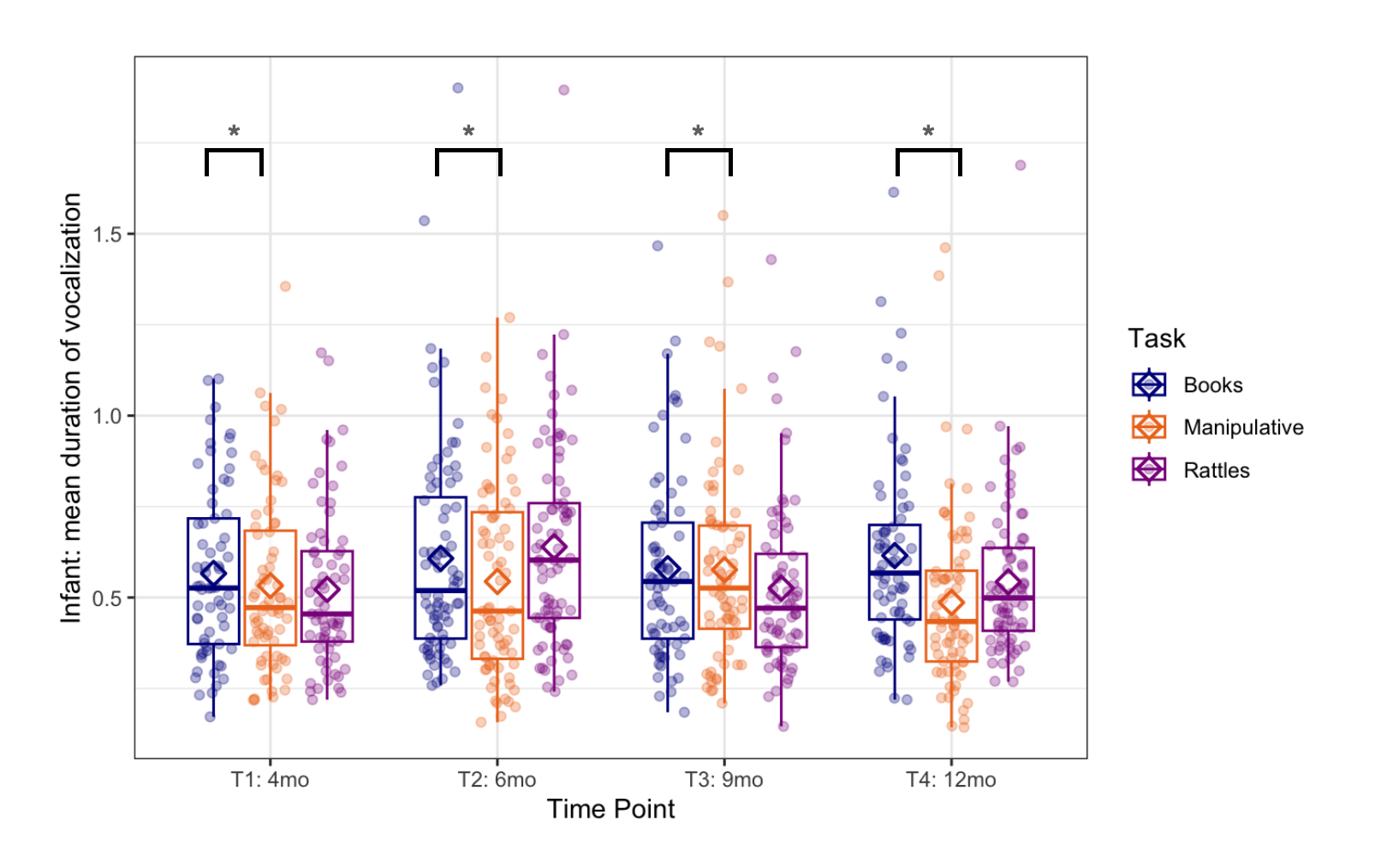


Fig. S3 Boxplots showing the mean duration of the infant's utterance at each time point during book-sharing (blue), playing with manipulative toys (orange), and rattle-shaking (purple). Horizontal lines represent the median value, boxes are drawn from the first quartile to the third quartile, and whiskers indicate min and max values. Diamonds represent mean scores. Significant differences indicated by asterisks: ***, *p < .001.*

The GEE with time point (4) and task (3) as within-subjects factors showed the main effects of task (Wald χ2 (2) = 6.89, p = 0.032) in the mean duration of the infant's vocalization. The mean duration of the infant's vocalization was longer during book-sharing than during playing with manipulative toys (p = 0.02). Neither the effect of time point (Wald χ2(3) = 6.05, p = 0.109) nor the interaction effect (Wald χ2(6) = 9.95, p = 0.127) were significant.


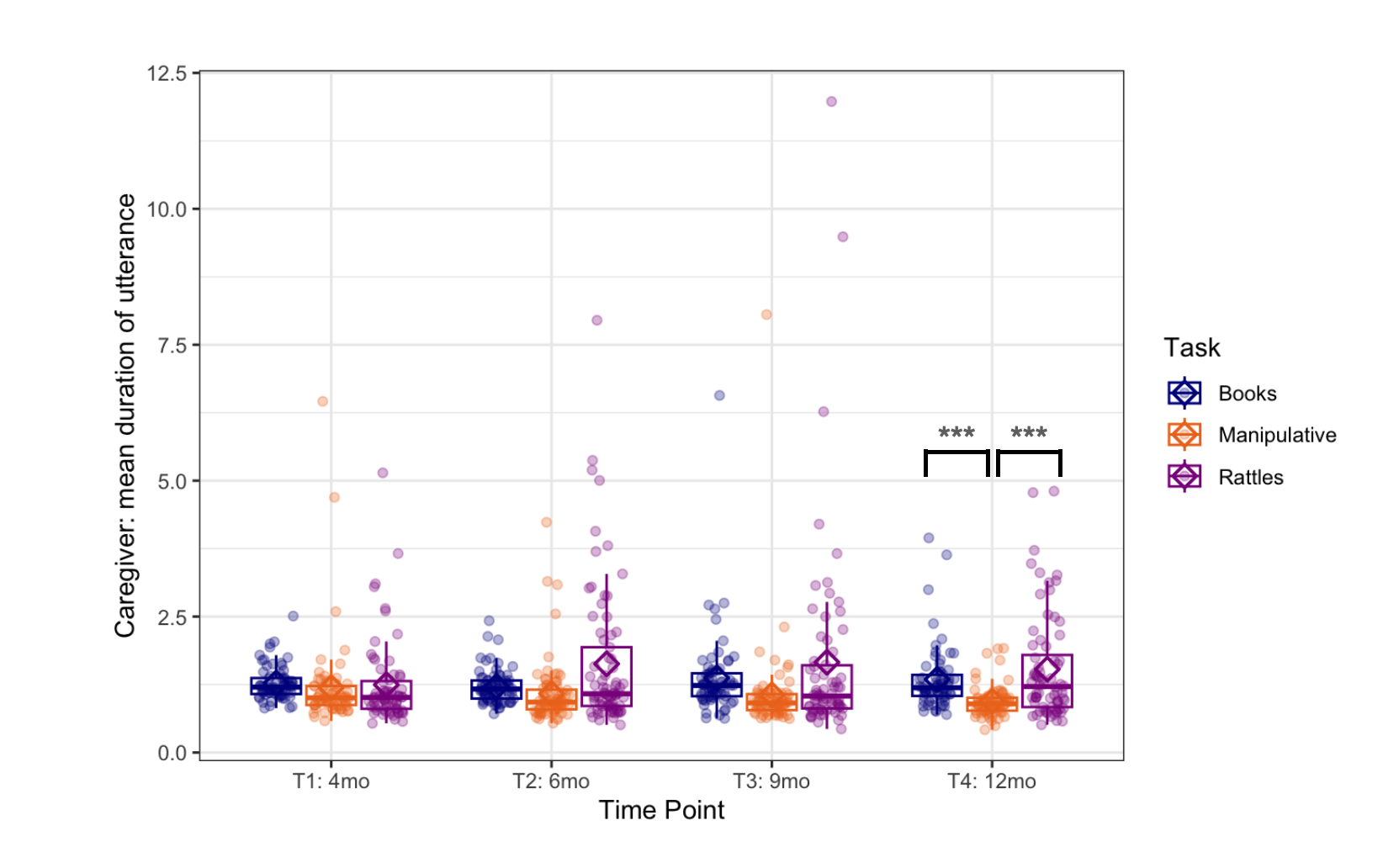


Fig. S4 Boxplots showing the mean duration of caregiver’s utterance at each time point during book-sharing (blue), playing with manipulative toys (orange), and rattle-shaking (purple). Horizontal lines represent the median value, boxes are drawn from the first quartile to the third quartile, and whiskers indicate min and max values. Diamonds represent mean scores. Significant differences indicated by asterisks: ***, *p < .001.*

The GEE with time point (4) and task (3) as within-subjects factors showed the main effects of task (Wald χ2 (2) = 34.3, p < 0.001) as well as an interaction task x time point (Wald χ2(6) = 17.4, p = 0.008) in the mean duration of caregiver's utterance. There was no main effect of time point (Wald χ2 (3) = 1.8, p = 0.614). At T4, the mean duration of the caregiver's utterance was shorter during playing with manipulative toys than during book-sharing (p < 0.001) or rattle-shaking (p < 0.001). The difference between book-sharing and rattle-shaking was not significant.

1. **Linear Mixed Models**

5.1. Rate per minute of infant vocalizations (Model: Rate_per_min_Speechlike ~ Time_Point * Task + (1|ID))


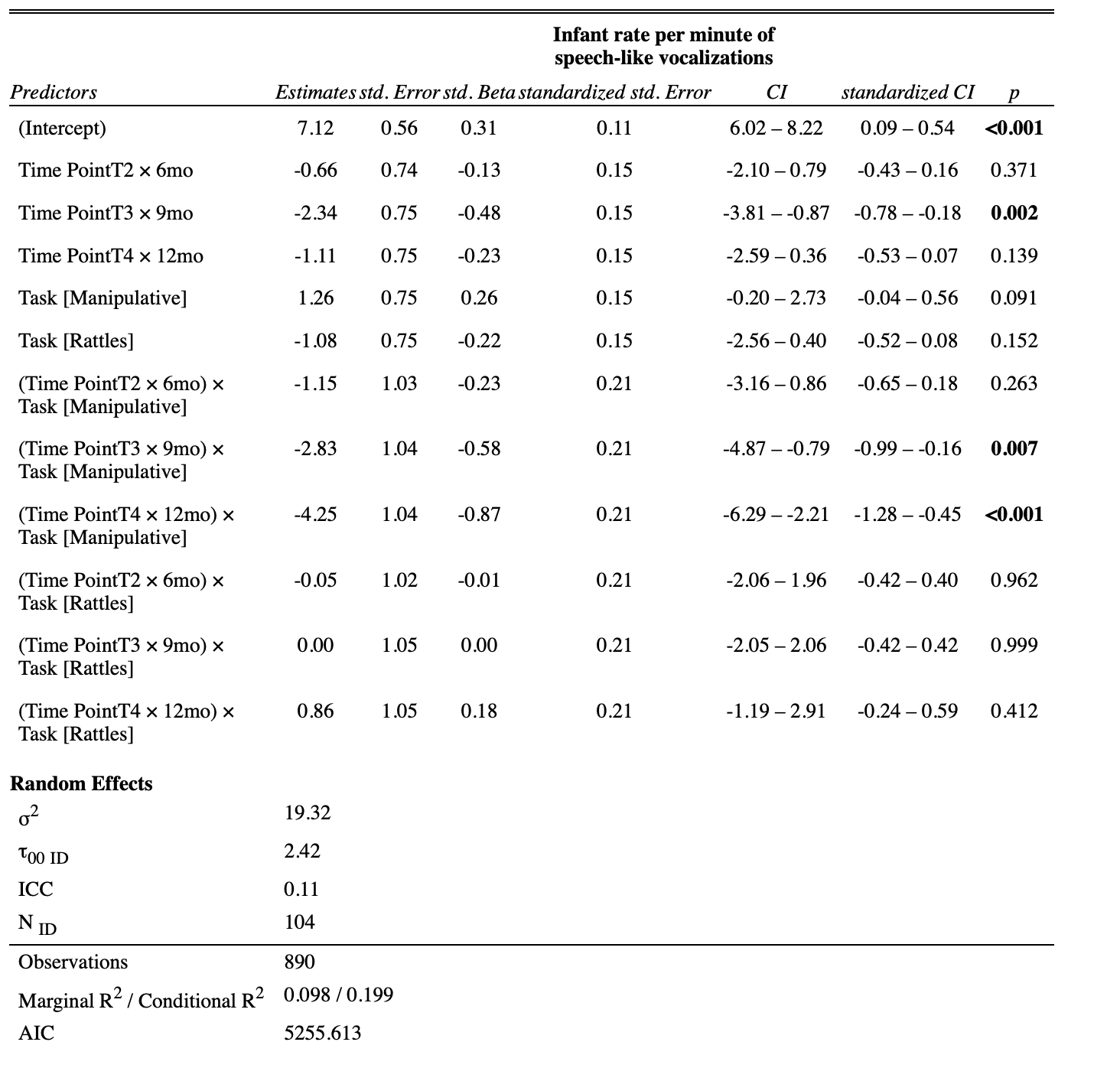


5.2. Rate per minute of caregiver vocal production (Model: CG_Rate_per_min_all_categories ~ Time_Point * Task + (1|ID))


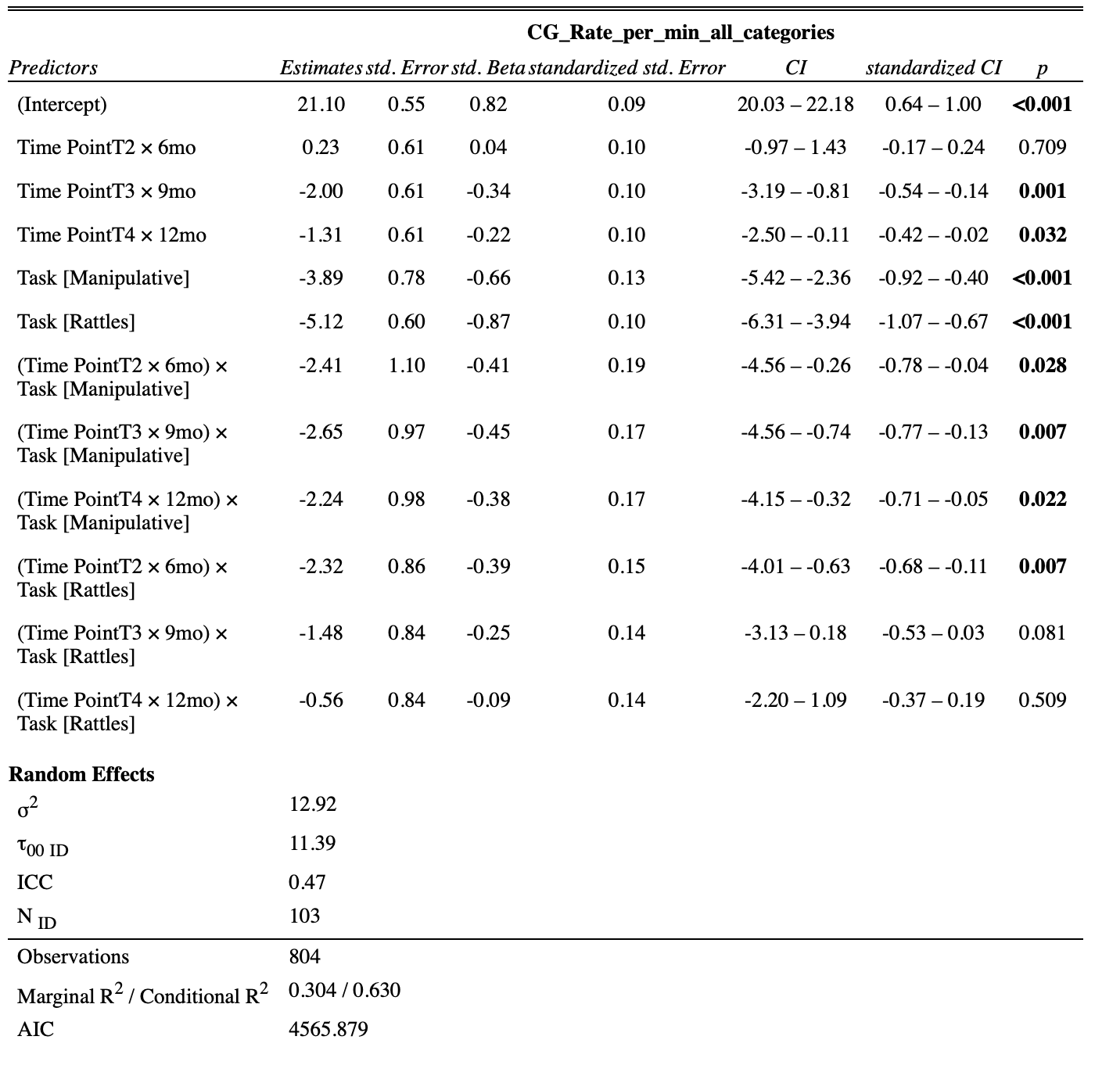


5.3. Dyadic vocal coordination

5.3.1.1 Rate per minute of conversational turns - infant’s responses to caregiver’s utterances (Model: TT_Inf_Transitions_count ~ Time_Point * Task + (1|ID))


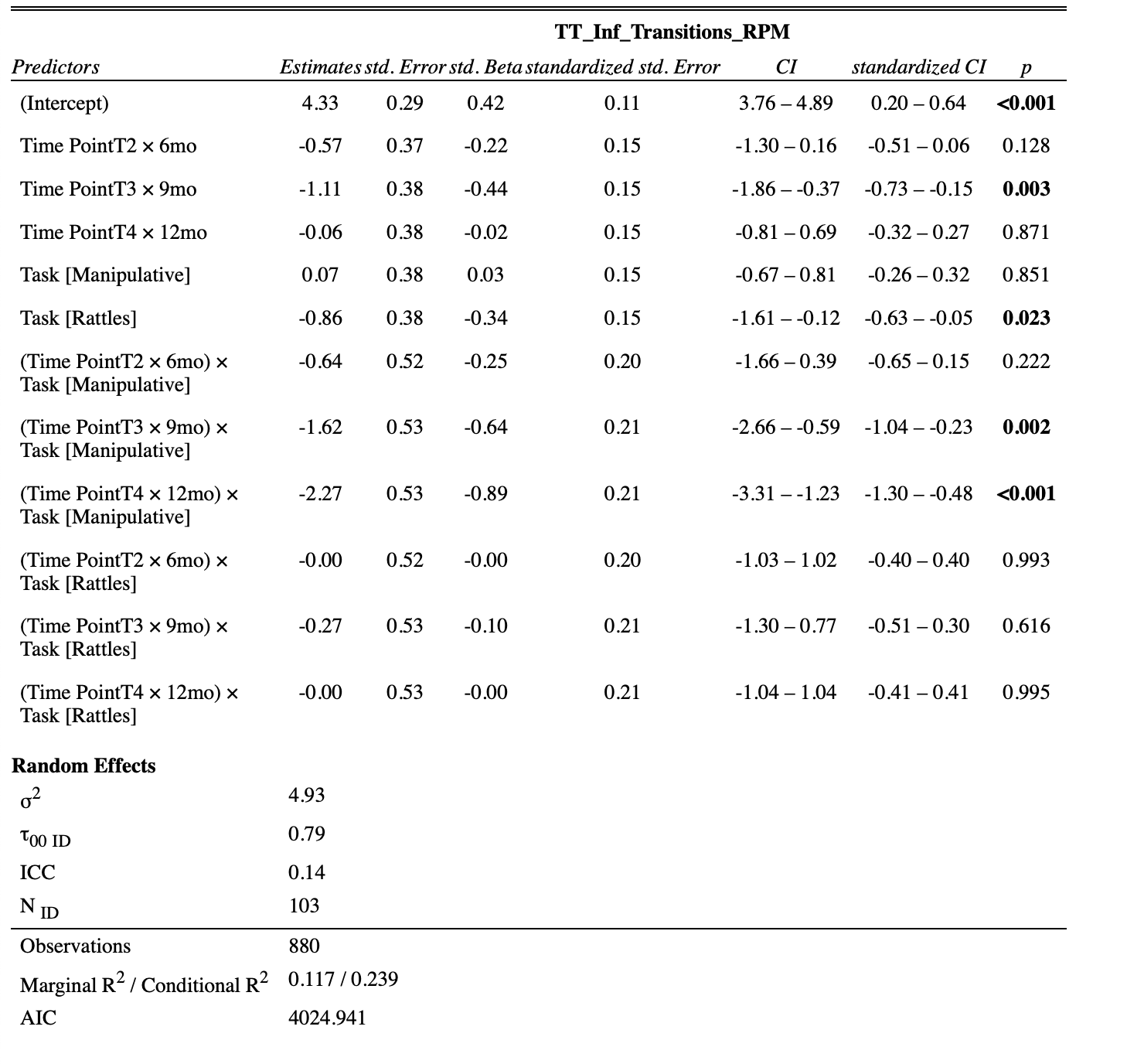


5.3.1.2. Rate per minute of conversational turns - caregiver’s responses to infant’s utterances (Model: TT_CG_Transitions_count ~ Time_Point * Task + (1|ID))
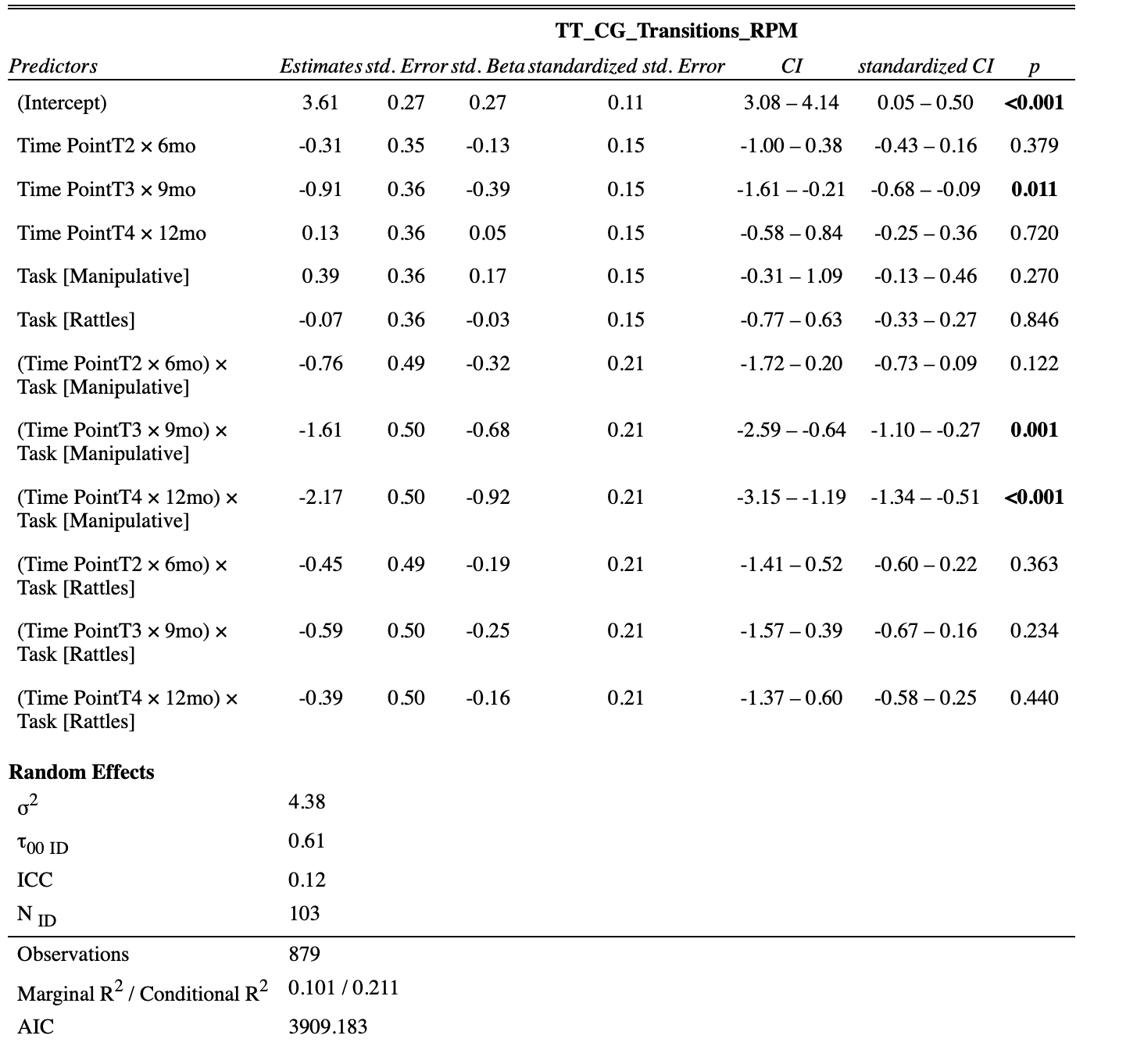


5.3.2.1. Mean turn transition time (gaps and overlaps) - infant's responses to the caregiver’s utterances (Model: TT_Inf_Transitions_mean ~ Time_Point * Task + (1|ID))


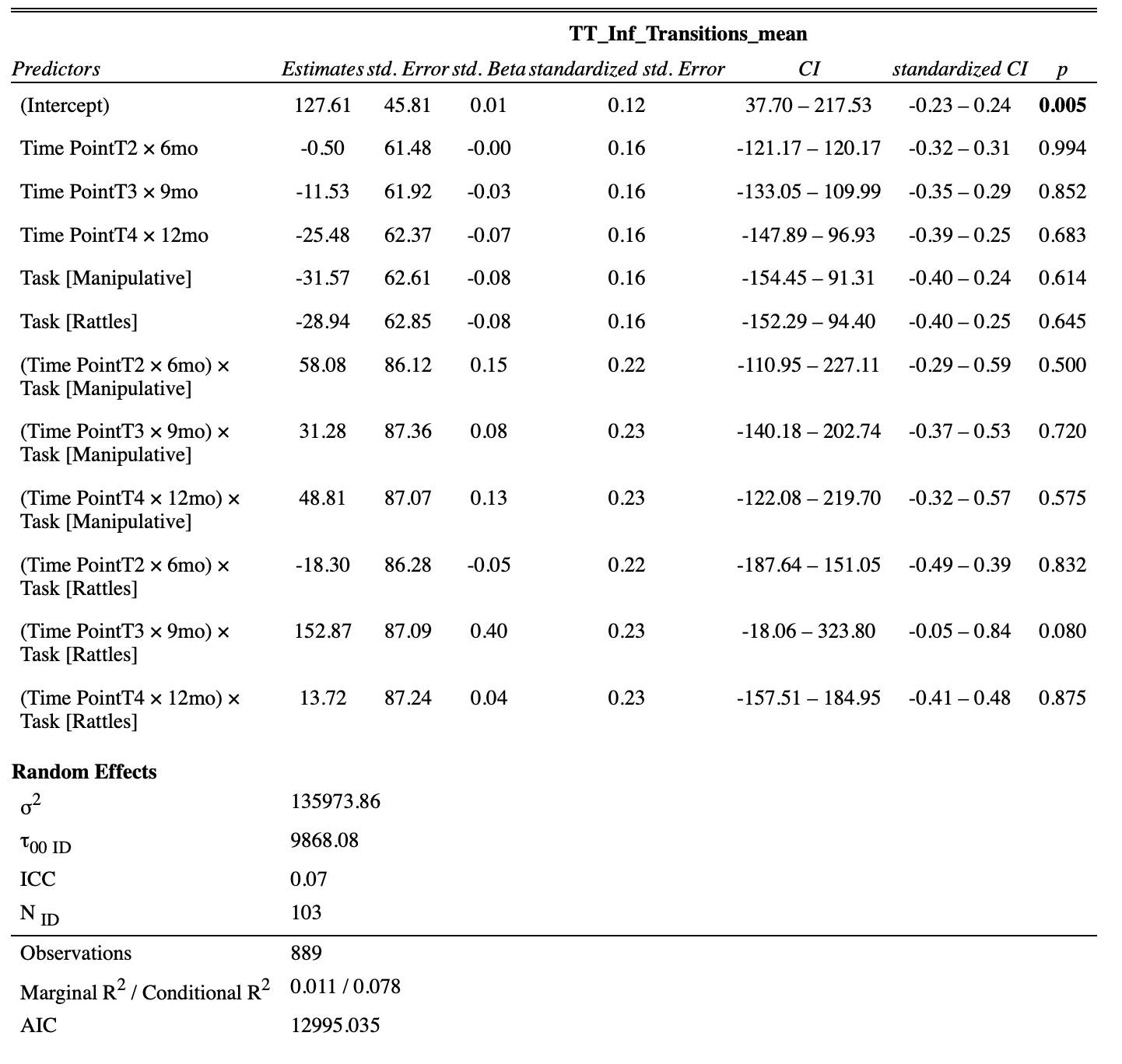


5.3.2.2. Mean turn transition time (gaps and overlaps) - caregiver's responses to the infant’s utterances (Model: TT_CG_Transitions_mean ~ Time_Point * Task + (1|ID))


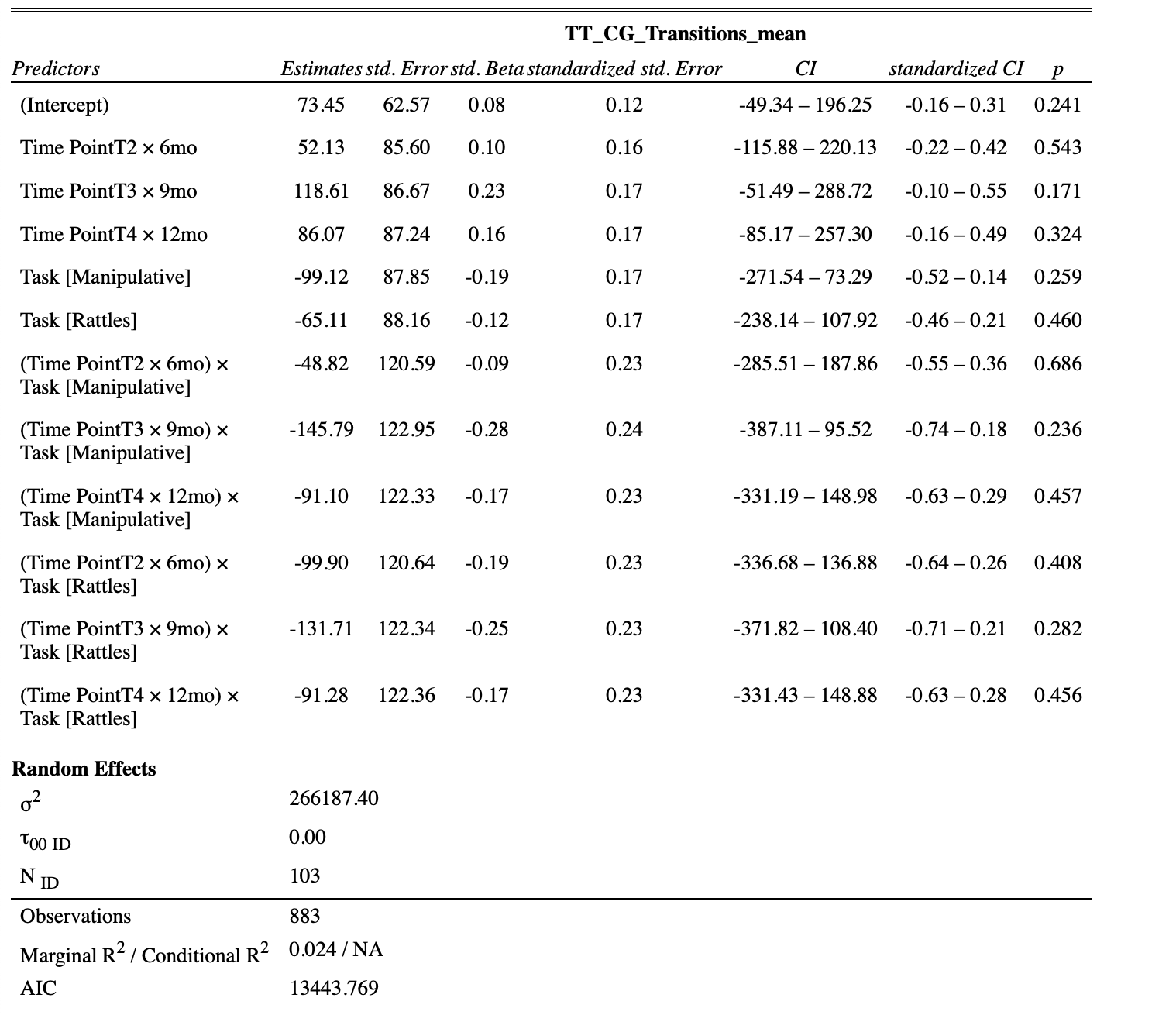


5.4. Mean duration of vocalization or utterance

5.4.1. Mean duration of infant speechlike vocalizations (Model: Mean_duration_Speechlike ~ Time_Point * Task + (1|ID))

**
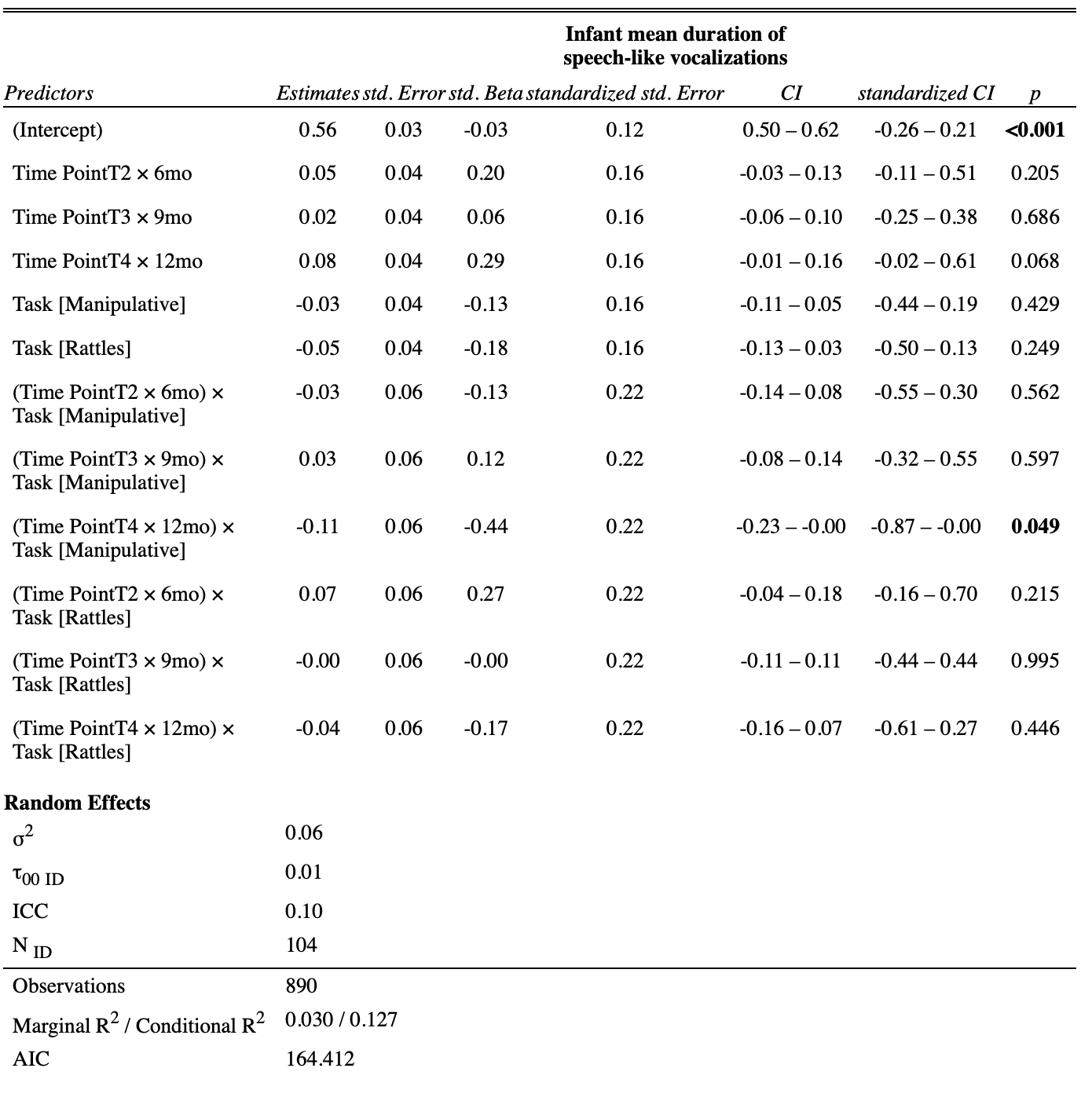
**

5.4.2. Mean duration of caregiver’s utterance (Model: CG_Mean_duration_all_categories ~ Time_Point * Task + (1|ID))

**
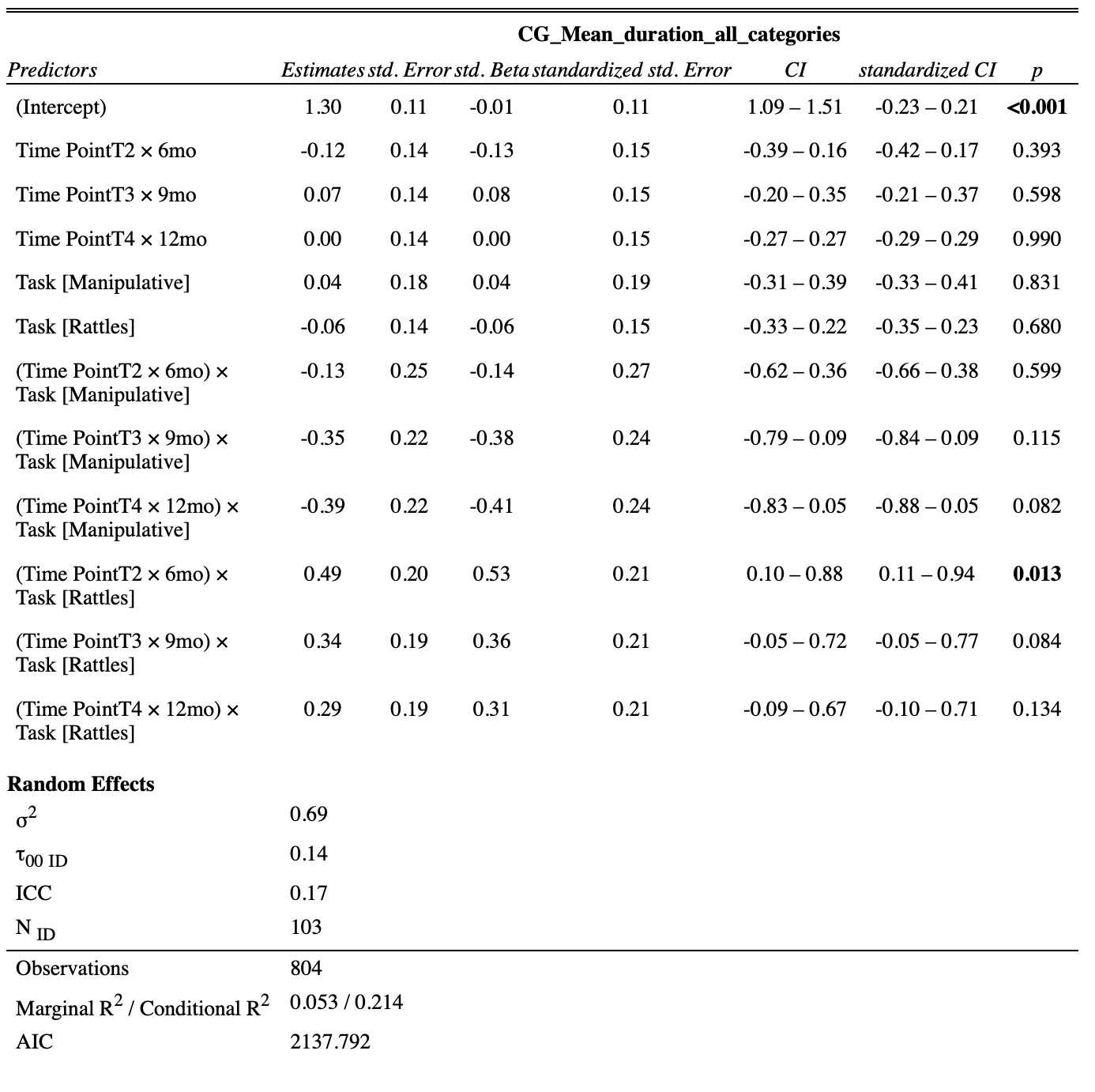
**

**6. Descriptive statistics**

|  | *Book-sharing* | | | *Playing with manipulative toys* | | | *Rattle-shaking* | | |
| --- | --- | --- | --- | --- | --- | --- | --- | --- | --- |
| Variable | *M* | *SD* | *Range* | *M* | *SD* | *Range* | *M* | *SD* | *Range* |
| Rate per minute of infant vocalizations at 4 months | 7.15 | 6.65 | [0.34; 25.57] | 8.40 | 7.76 | [0.19; 34.40] | 5.99 | 5.29 | [0.38; 26.67] |
| Rate per minute of infant vocalizations at 6 months | 6.49 | 4.96 | [0.16; 20.13] | 6.62 | 5.57 | [0.36; 26.74] | 5.38 | 4.08 | [0.19; 20.51] |
| Rate per minute of infant vocalizations at 9 months | 4.93 | 3.64 | [0.19; 16.27] | 3.38 | 3.12 | [0.17; 18.57] | 3.82 | 2.76 | [0.19; 13.70] |
| Rate per minute of infant vocalizations at 12 months | 6.14 | 3.68 | [0.54; 18.30] | 3.12 | 2.26 | [0.19;  9.48] | 5.91 | 3.75 | [0.19; 18.04] |
| Rate per minute of caregiver vocal production at 4 months | 21.23 | 4.31 | [9.47; 33.43] | 17.28 | 4.82 | [6.13; 26.69] | 16.15 | 5.32 | [1.86; 26.94] |
| Rate per minute of caregiver vocal production at 6 months | 21.24 | 4.37 | [8.88; 31.66] | 15.30 | 5.95 | [3.83; 28.35] | 13.92 | 4.42 | [5.51; 23.92] |
| Rate per minute of caregiver vocal production at 9 months | 18.73 | 5.01 | [0.39; 29.51] | 12.28 | 5.00 | [1.35; 21.76] | 12.15 | 5.45 | [0.38; 23.56] |
| Rate per minute of caregiver vocal production at 12 months | 19.67 | 3.92 | [7.48; 28.67] | 13.65 | 5.56 | [0.80; 26.32] | 13.95 | 4.87 | [1.89; 25.66] |
| Rate per minute of conversational turns (Infant to caregiver) at 4 months | 4.38 | 3.55 | [0.19; 12.98] | 4.46 | 3.37 | [0.19; 13.71] | 3.49 | 2.82 | [0.34; 11.65] |
| Rate per minute of conversational turns (Infant to caregiver) at 6 months | 3.76 | 2.54 | [0.16; 11.19] | 3.22 | 2.50 | [0.19; 9.45] | 2.93 | 2.04 | [0; 9.52] |
| Rate per minute of conversational turns (Infant to caregiver) at 9 months | 3.26 | 2.29 | [0; 11.02] | 1.73 | 1.32 | [0; 5.65] | 2.12 | 1.60 | [0.19; 7.83] |
| Rate per minute of conversational turns (Infant to caregiver) at 12 months | 4.33 | 2.26 | [0.18; 10.0] | 2.10 | 1.65 | [0.18;  7.08] | 3.45 | 2.07 | [0; 9.61] |
| Rate per minute of conversational turns (Caregiver to infant) at 4 months | 3.7 | 3.0 | [0; 11.02] | 4.05 | 3.18 | [0.18; 12.82] | 3.55 | 2.96 | [0.34; 13.94] |
| Rate per minute of conversational turns (Caregiver to infant) at 6 months | 2.23 | 3.31 | [0.16; 10.55] | 2.96 | 2.43 | [0.17;  9.57] | 2.81 | 1.95 | [0.15; 7.66] |
| Rate per minute of conversational turns (Caregiver to infant) at 9 months | 3.26 | 2.29 | [0; 11.02] | 1.54 | 1.16 | [0; 5.44] | 2.07 | 1.73 | [0; 8.70] |
| Rate per minute of conversational turns (Caregiver to infant) at 12 months | 3.80 | 2.03 | [0; 9.75] | 1.99 | 1.55 | [0; 7.22] | 3.34 | 1.98 | [0.56; 9.87] |
| Mean turn transition time (Infant to caregiver) at 4 months | 124.7 | 377.6 | [-489.0; 2298.0] | 92.47 | 490.66 | [-2982.0; 1709.5] | 96.8 | 407.13 | [-1485.5; 1089.5] |
| Mean turn transition time (Infant to caregiver) at 6 months | 128.13 | 259.82 | [-493.44; 857.0] | 155.40 | 328.23 | [-510.0;  1851.0] | 83.17 | 332.54 | [-940.0;  1197.0] |
| Mean turn transition time (Infant to caregiver) at 9 months | 122.08 | 335.14 | [-558.33; 1393.33] | 120.77 | 474.03 | [-825.5; 2210.0] | 245.04 | 479.72 | [-536.94; 2391.0] |
| Mean turn transition time (Infant to caregiver) at 12 months | 110.18 | 224.53 | [-620.0; 1032.33] | 123.77 | 477.64 | [-1776.0; 1660.5 | 92.21 | 317.26 | [-927.37; 830.71] |
| Mean turn transition time (Caregiver to infant) at 4 months | 73.45 | 285.97 | [-924.0; 1020.17] | -25.67 | 563.08 | [-2467.0; 2175.0] | 8.34 | 469.65 | [-1324.5; 1700.5] |
| Mean turn transition time (Caregiver to infant) at 6 months | 125.58 | 418.99 | [-1919.0; 1125.75] | -22.37 | 554.90 | [-1851.0; 1491.0] | -39.43 | 561.00 | [-1410; 2325] |
| Mean turn transition time (Caregiver to infant) at 9 months | 192.07 | 460.07 | [-499.10; 2317.67] | -52.84 | 495.7 | [-1565.0; 1945.0] | -4.75 | 695.24 | [-2455.0; 1869.0] |
| Mean turn transition time (Caregiver to infant) at 12 months | 159.52 | 376.49 | [-1038.75; 1468.33] | -30.71 | 720.08 | [-2969.0; 2692.0] | 3.13 | 444.04 | [-1450.33; 936.0] |
|  |  |  |  |  |  |  |  |  |  |

*Note: M =* mean, *SD =* standard deviation.
